# Effects of Kinesio tape on lower limb muscle strength, hop test, and vertical jump performances: a meta-analysis

**DOI:** 10.1186/s12891-019-2564-6

**Published:** 2019-05-14

**Authors:** Ming Lok Yam, Zuyao Yang, Benny Chung-Ying Zee, Ka Chun Chong

**Affiliations:** 1 JC School of Public Health and Primary Care, Faculty of Medicine, The Chinese University of Hong Kong, The Jockey Club School of Public Health Building, Prince of Wales Hospital, Shatin, New Territories Hong Kong, China; 2 Shenzhen Research Institute, The Chinese University of Hong Kong, Shenzhen, China

**Keywords:** Muscle fatigue, Knee osteoarthritis, Patellofemoral pain syndrome, Anterior cruciate ligament reconstruction, Muscle strength, Functional performance

## Abstract

**Background:**

To date, published systematic reviews concerning the effects of Kinesio Taping (KT) on muscle strength have not analysed facilitatory and inhibitory applications separately. As a result, their results could be substantially affected by clinical heterogeneity. This meta-analysis was conducted to determine the effectiveness of using a facilitatory application of KT for lower limb muscle strength and functional performance (distance in a single-leg hop and vertical jump height) in individuals without disabilities and in those with musculoskeletal conditions (muscle fatigue, chronic musculoskeletal diseases, and post-operative orthopaedic conditions).

**Methods:**

Searches were conducted on six major electronic databases. Randomised controlled trials that used facilitatory KT were included. Standardised mean differences (SMDs) were calculated and random-effects models were used for analysis.

**Results:**

Thirty-seven randomised controlled trials were included. KT was superior to controls for improving lower limb muscle strength in individuals with muscle fatigue (short-term effect, pooled SMD = 0.53, 95% CI = 0.09 to 0.96; long-term effect, pooled SMD = 0.61, 95% CI = 0.12 to 1.11) and in individuals with chronic musculoskeletal diseases (pooled SMD = 1.24, 95% CI = 0.33 to 2.16) with large effect sizes. The use of KT in populations without disabilities was not supported. There is insufficient evidence for the effect of KT on functional performance in individuals with musculoskeletal conditions.

**Conclusions:**

Contrary to prior research, the existing evidence shows that KT can improve lower limb muscle strength in individuals with muscle fatigue and chronic musculoskeletal diseases. The effect sizes produced in this meta-analysis show that KT may be superior to some existing treatments for these conditions. In addition, this study suggests that practitioners may wish to avoid the use of KT in individuals without disabilities.

**Trial registration:**

PROSPERO registration number CRD42017075490, registered on 21 November 2017.

**Electronic supplementary material:**

The online version of this article (10.1186/s12891-019-2564-6) contains supplementary material, which is available to authorized users.

## Background

Kinesio tape (KT) is a commonly used adhesive elastic tape. The hypothesised effects of KT include reduced pain, facilitated or inhibited muscle strength, and increased range of motion [1]. The manufacturer claims that KT can facilitate muscle contractions if it is applied from the origin of the muscle to its insertion point and that KT can inhibit muscle contractions if it is applied from the insertion point to the origin of the muscle [1]. One of the proposed mechanisms is that the recoiling force of KT may be transmitted to the fascia [2]. This force may then assist in muscle contractions if the contraction and the KT have the same direction of pull. [3] In contrast, the pulling force may weaken muscle contractions if the KT and the muscle contraction have opposite directions of pull. Another proposed mechanism is that the ability of KT to recoil may stimulate cutaneous mechanoreceptors [4]. This effect would increase motor unit excitability and elicit a muscle spindle reflex if the direction of pull matches the direction of muscle contraction. KT’s pulling force may also stretch the Golgi tendon organs if the directions of the pull and the muscle contraction are in opposite directions. In this case, KT would inhibit muscle contraction. It has also been claimed that because KT can be kept on the skin for 3–5 days, KT can provide prolonged treatment [5].

There are few meta-analyses investigating the treatment effects of KT [6–10]. A meta-analysis conducted by Chang et al. reported that KT can relieve pain and increase the flexibility of muscles in patients with patellofemoral pain syndrome [6]. Lu et al. indicated that KT is effective for reducing pain and improving knee flexion range of motion in patients with knee osteoarthritis [7]. Parreira et al. reported that the research findings published through June 2013 did not support the effects of KT on pain, disability, quality of life, return to work, or global impression of recovery [8]. However, the meta-analysis from Parreira et al. included very few studies and was thus limited in its statistical power. Lim and Tay conducted a meta-analysis and summarised the literature published through July 2014 [9]. They reported that KT is superior to placebo or no tape for chronic musculoskeletal pain relief. Csapo and Alegre conducted a meta-analysis examining the effects of KT on skeletal muscle strength [10]. They included studies published through March 2014. Their study results did not support an enhancement in muscle strength from KT in subjects without disabilities. However, the abovementioned meta-analyses have several limitations. First, they did not analyse the use of facilitatory and inhibitory KT separately. As mentioned above, these two applications may have different physiological mechanisms. In addition, Lim and Tay indicated that differences in taping direction may alter the outcome measures [9]. Because previous meta-analyses did not take potential clinical heterogeneity among the included studies into account, the study results may be distorted. Second, the study by Csapo and Alegre included non-randomised controlled trials [10], which provide weaker evidence compared to randomised controlled trials. Third, the four abovementioned meta-analyses only included papers published up to 2014 [6, 8–10]. From 2014 to 2018, a large number of KT-related randomised controlled trials were published (see in Additional file 2 :Tables S1-S4 [11–38]. Fourth, the previous meta-analyses did not investigate the effects of KT in populations with other musculoskeletal conditions, such as muscle fatigue and post-operative orthopaedic conditions, even though KT is widely used as a treatment for these two conditions [14, 15, 35–39]. Therefore, an updated meta-analysis of randomised controlled trials examining the effects of KT on muscle strength is warranted to better summarise these emerging studies. This study avoids potential heterogeneity and will investigate populations with different musculoskeletal conditions.

The objectives of this systematic review and meta-analysis of randomised controlled trials were as follows: 1) to compare the effects of facilitatory KT with other interventions for improving lower limb muscle strength (primary outcome) and 2) to compare the effects of facilitatory KT with other interventions for improving lower limb functional performance test results (secondary outcomes) in individuals without disabilities and in those with musculoskeletal conditions (muscle fatigue, chronic musculoskeletal diseases, and post-operative orthopaedic conditions). Only facilitatory KT was analysed to eliminate potential clinical heterogeneity. For the meta-analyses mentioned above [6–10], more studies pertaining to lower limbs were included compared to those concerning upper limbs. Therefore, to reduce the heterogeneity between studies, this meta-analysis only included studies examining lower limbs. Functional performance test results were included to provide evidence for clinical applications of KT. Indeed, several studies have investigated the effects of KT on lower limb functional performance. For instance, Bicici et al. conducted a study that measured performance in several lower limb functional tests after applying KT [40]. KT was shown to be superior to placebo and no tape in improving single limb-hurdle test results. The present study included distance in a single-leg hop and vertical jump height tests for analyses since these two tests were most commonly studied. [41] This meta-analysis was registered with PROSPERO (registration number CRD42017075490).

## Methods

### Information sources

This study was conducted following the Preferred Reporting Items for Systematic Reviews and Meta-Analyses (PRISMA) guidelines. Literature searches were conducted using EMBASE, MEDLINE, CINAHL, Cochrane Central Register of Controlled Trials (CENTRAL), the Physiotherapy Evidence Database (PEDro), and Google Scholar. The reference lists of the included studies and related systematic reviews were also searched. The searches were limited to “human”, “English language”, and “randomised controlled trial”. The keywords used were “Kinesio taping”, “strength”, “function”, “performance”, and their synonyms. Examples of the search strategies for EMBASE, MEDLINE, CINAHL, and CENTRAL are shown in the Additional file 2: Tables S6-S9. The last search was performed on 1 February 2018.

### Eligibility criteria

This meta-analysis included all published randomised controlled trials with both parallel and crossover designs that compared the effects of facilitatory KT with a control (sham taping/placebo taping/conventional treatment). Only KT applied from the muscle origin to the insertion point was defined as facilitatory KT (e.g., from 10 cm below the anterior superior iliac spine to the base of the patella for the rectus femoris muscle). Studies with KT applied from the muscle insertion to the origin and those using a fan-shape application (for lymphatic drainage purposes) were excluded. This study did not restrict the age, race, or physical activity level of the included study populations or the brand of KT. This study included populations without disabilities as well as those with muscle fatigue, chronic musculoskeletal diseases, and post-operative orthopaedic conditions. Chronic musculoskeletal disease was defined as symptoms lasting longer than 4 weeks [42]. Studies that only assessed upper limbs and studies that investigated populations with neurological diseases were excluded. Studies were only included if they had at least one of the following outcome measures: lower limb muscle strength, distance in a single-leg hop, or vertical jump height. Only studies published in English between 2007 and 2018 were included.

A checklist of the inclusion and exclusion criteria was generated to standardise the screening process. Study titles and abstracts were screened first. Full texts of the studies were evaluated if necessary. Two independent researchers assessed the eligibility of each study (MLY and KCC). Any disagreements between the two reviewers were resolved by consensus.

### Data extraction and quality assessment

A standardised data extraction form was used for data collection. The study design (parallel or crossover), study population characteristics (number of participants, age, and sex), disease status (type of chronic musculoskeletal disease, surgery performed, and post-intervention period), intervention details (application site, shape of tape, and tension applied), control details (type of tape used, taping method), and outcome measures (lower limb muscle strength, single-leg hop test result or vertical jump height) were extracted. For crossover trials, washout periods were recorded to investigate potential carry-over effects. For subjects with muscle fatigue and without disabilities, both the short-term and long-term effects of KT were investigated. A short-term KT application outcome was defined as the earliest post-intervention value with KT in situ for less than 24 h; a long-term KT application outcome was defined as the last post-intervention value with KT in situ for at least 24 h. For studies in individuals with muscle fatigue, only the post-fatigue protocol outcomes were extracted for analysis. The cut-off was set at 24 h because Słupik et al. have reported that the bioelectrical activity of KT is increased after a 24-h application [43]. For the studies investigating populations with chronic musculoskeletal diseases and post-operative orthopaedic conditions, only the final post-intervention value was extracted to assess the effectiveness of KT on lower limb function recovery. For studies reporting the strength of multiple muscles, only knee extension strength was extracted because it was the most-often reported parameter among the included studies. If both concentric and eccentric muscle strengths were measured, concentric muscle strength was extracted since most included studies measured concentric strength. Some studies only measured isometric muscle strength; however, for studies that measured isokinetic muscle strength with multiple angular velocities, only the isokinetic strength measured with the lowest angular velocity was extracted as it is more comparable to isometric muscle strength than measurements made with higher angular velocities [44]. For studies in which KT was applied using multiple tensions, the tension closest to that recommended by Kase et al. for muscle facilitation (25 to 50%) was selected. [45] For studies that included both placebo/sham taping and no-tape groups as control groups, the outcomes of the placebo/sham taping group were selected to eliminate placebo effects.

The study quality was assessed using the PEDro scale. PEDro scores were retrieved from the PEDro database when available. For studies without corresponding scores in the PEDro database, the articles were assessed by the author who had completed the PEDro scale training programme (MLY). The PEDro scale rates the study quality using 11 items. The score is calculated by summing the number of items that the evaluated study meets. The first item does not assess internal validity; therefore, it is not included in the total score calculation. The total score ranges from 0 to 10. Scores of 9–10, 6–8, 4–5, and 0–3 indicate “excellent”, “good”, “fair”, and “poor” quality, respectively [46].

### Data synthesis and analysis

The meta-analyses were performed using RevMan 5 (The Cochrane Collaboration, Copenhagen, Denmark). Standardised mean differences (SMDs) and standard errors (SEs) were calculated for the different outcome measures. Specifically, for crossover trials, the SMDs and SEs were calculated using the formulae (KT mean − control mean)/[(KT SD [2] (standard deviation) + control SD [2])/2]^1/2^ and (1/N + SMD [2]/2 N)^1/2^ × [2(1 – correlation coefficient)]^1/2^, respectively. For studies providing SDs of within-participant differences (WDs), the correlation coefficient was computed using the formula (KT SD [2] + control SD [2] –WD SD^2^)/(2 × KT SD [2] × control SD [2]). The correlation coefficients of all studies providing SDs of WDs were calculated, and the median available inter-trial correlation coefficient was obtained. For some crossover studies in which the correlation coefficient could not be calculated, the median available inter-trial correlation coefficient was used to obtain the SMD and SE. Random-effects models were used to calculate pooled estimates due to potential clinical heterogeneity among the included studies. SMDs of 0.2, 0.5, and 0.8 were defined as small, moderate, and large effect sizes (ESs), respectively [47]. An SMD smaller than 0.2 was defined as a trivial ES. Planned subgroup analyses were conducted for studies that investigated populations with post-operative orthopaedic conditions by splitting the studies into the following two groups: one group for the acute setting and another group for the non-acute setting. The acute group had post-operative periods no longer than six weeks, while the non-acute group had post-operative periods greater than six weeks [48]. Planned sensitivity analyses were performed by excluding crossover trials that did not report washout periods and studies with poor quality scores (PEDro scores 0–3). Heterogeneity was assessed using the I^2^ statistic. Publication bias was assessed using Egger’s test if the number of studies included in the meta-analysis was greater than 10. If the number of included studies was less than 10, then publication bias was assessed via visual inspection of the funnel plots and the trim-and-fill method. If publication bias was present, then post hoc sensitivity analyses were conducted using the trim-and-fill method. Publication bias tests were conducted using the metafor package in R version 3.4.4 (R Foundation for Statistical Computing, Vienna, Austria).

## Results

### Study selection

**Fig. 1:**
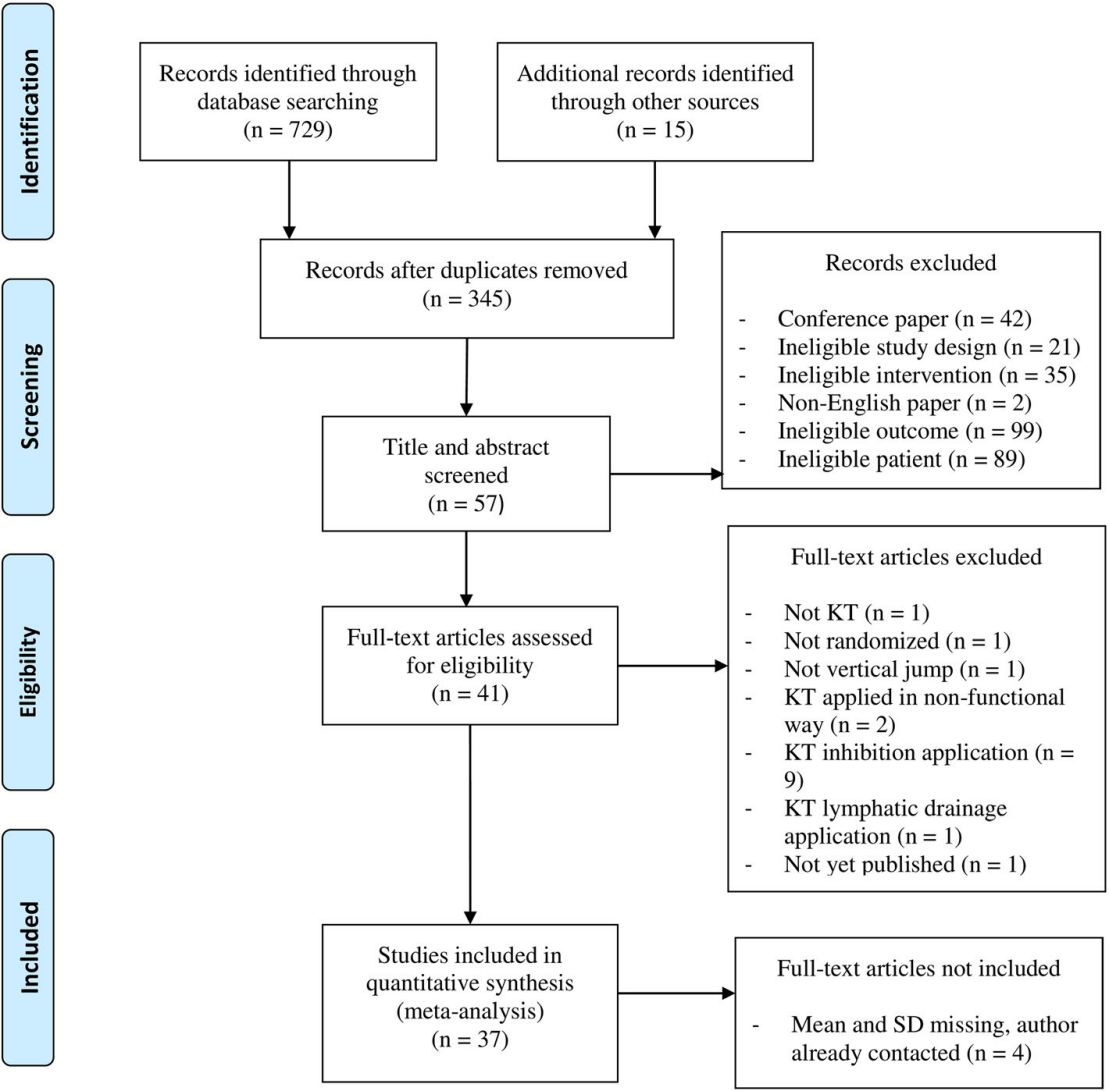
Flow Diagram for study selection [76]

The initial search on the electronic databases identified 729 studies, and 15 additional articles were identified after a manual search. After screening, 5 studies were found to have missing data for the required variables (e.g., mean and SD of the outcomes) [36, 49–52]. The corresponding authors were contacted via email, but only one author replied. [36] As a result, 37 studies were considered eligible after screening [2, 4, 11–39, 53–58]. The detailed selection process is shown in Fig. 1. Details of the included studies are summarised in in the Additional file 2: Tables S1-S4.

### Methodological quality and study characteristics

The assessment details of the PEDro scale are shown in the Additional file 2 : Table S5. The average PEDro score of the included studies was 5.16 (1.65) [mean (SD)]. No studies had therapist blinding as it is not possible in a taping intervention. Other common sources of bias in the included studies were as follows: 1) lack of intention-to-treat analyses (89.2%), 2) lack of allocation concealment (70.3%), 3) lack of patient blinding (64.9%), and 4) lack of assessor blinding (64.9%). A total of 40.5% (15/37) of the studies used crossover designs. Among the included carry-over studies, the average washout period was 5.83 days (range 2–7). This range is considered adequate owing to the non-pharmacological properties of KT. However, three studies did not report using washout periods [4, 13, 16]. These studies were excluded from the planned sensitivity analysis.

Among the 15 studies that used crossover designs, coefficient correlations could only be calculated from 5 studies [4, 24, 55–57]. The median inter-trial correlation coefficients for lower limb muscle strength and vertical jump height were 0.89 and 0.94, respectively.

For the characteristics of the control groups, one study [39] that investigated the effect of KT on muscle strength in a population with muscle fatigue used an “add-on” design with active treatment as the control (KT + static stretching versus proprioceptive neuromuscular facilitation stretching + static stretching). Two studies [31, 32] were conducted in populations with chronic musculoskeletal diseases, and two studies [35, 36] were conducted in populations with post-operative orthopaedic conditions used an “add-on” design with minimal intervention as the control (KT + conventional therapy versus placebo/sham taping/no tape + conventional therapy). All other studies used only minimal intervention (placebo/sham taping/no tape) as a control. A post hoc sensitivity analysis was conducted by excluding the study that used an “add-on” design with active treatment as the control. The studies using an “add-on” design with minimal intervention as the control did not require a sensitivity analysis because the intervention comparison in these studies is theoretically similar to that of studies using only a minimal intervention as the control. To summarise, except for the meta-analysis for muscle strength in populations with muscle fatigue, the intervention comparisons of the other meta-analyses could be regarded as comparing KT versus minimal intervention.

Of the types of muscle contractions measured in the included studies, 29% of the included studies (9/31) measured only concentric strength, 52% (16/31) only measured isometric strength, 3% only (1/31) measured eccentric strength, and 16% (5/31) measured both concentric and eccentric strength.

### Lower limb muscle strength: populations with muscle fatigue

**Fig. 2:**
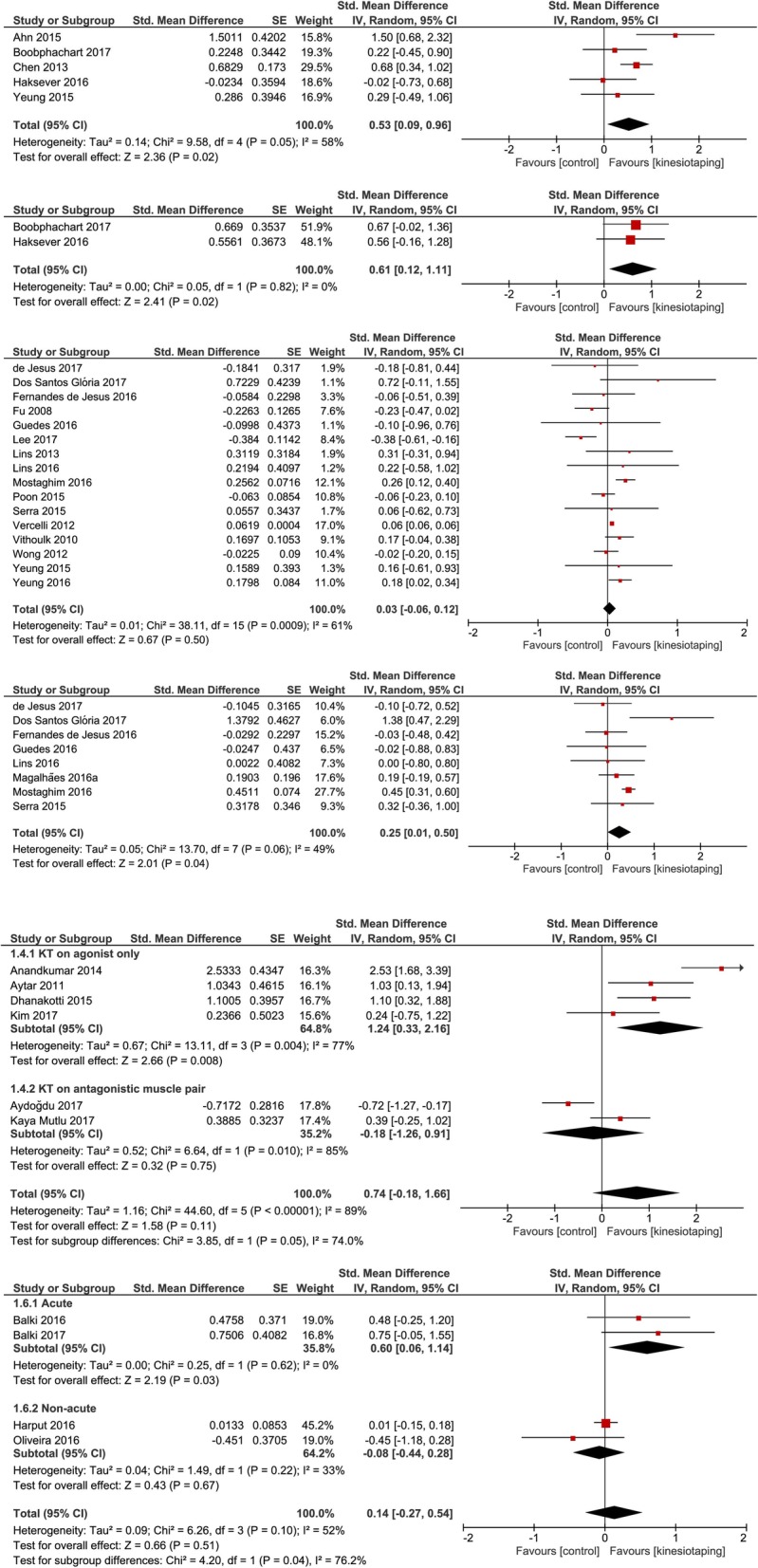
Meta-analyses for the effect of KT on muscle strength. The order of population groups from the top to the bottom (Short-term effect of KT in population with muscle fatigue, long-term effect of KT in population with muscle fatigue, short-term KT application in population without disabilities, long-term KT application in population without disabilities, population with chronic musculoskeletal diseases, and population with post-operative orthopaedic conditions)

In comparing the short-term effects of KT and the controls (including both active treatment and minimal intervention), the pooled SMD was significant (SMD = 0.53, 95% CI = 0.09 to 0.96; Fig. 2). The ES was moderate and favoured KT. The result may represent substantial heterogeneity (I^2^ = 58%). The heterogeneity was mainly due to the study from Ahn et al. [11] A post hoc sensitivity analysis was performed after excluding this study (in Additional file 1: Figure S23); the resulting pooled SMD was still significant (SMD = 0.40, 95% CI = 0.06 to 0.74) and the heterogeneity changed to “not important” (I^2^ = 27%). Another post hoc sensitivity analysis was performed after excluding the studies that used an “add-on” design with active treatment as the control (in Additional file 1: Figure S23) [39]. The pooled SMD between KT and minimal intervention was no longer significant (SMD = 0.47, 95% CI = − 0.16 to 1.10) but was still positive (favouring KT). It is notable that the heterogeneity increased after excluding this study (I^2^ = 65%). This factor contributed to the widening of the confidence interval. Moreover, a post hoc sensitivity analysis was conducted after excluding the two studies mentioned above (in Additional file 1: Figure S23). The pooled SMD was not significant (SMD = 0.16, 95% CI = − 0.26 to 0.57) but was still positive (favouring KT). It is noted that excluding these two studies decreased the sample size and widened the confidence interval. In addition, a post hoc subgroup analysis was conducted by separating studies that measured concentric, isometric, and eccentric contractions into different subgroups (in Additional file 1: Figure S24). No significant heterogeneity between the three subgroups was detected (*P* = 0.20). Therefore, it is unlikely that the difference in muscle strength measurements (concentric, isometric and eccentric) affected the outcome of the present analysis. Only two studies measured the long-term effects of KT (Fig. 2) [12, 14]. The result was significant (SMD = 0.61, 95% CI = 0.12 to 1.11), favoured KT, and had a moderate ES. This result should be interpreted with caution as only 2 studies were included. However, it is notable that the heterogeneity was very low (I^2^ = 0%), and the outcome matched the result of short-term effects. A post hoc subgroup analysis was conducted by separating studies that measured isometric and eccentric contractions into different subgroups (in Additional file 1: Figure S25). No significant heterogeneity was detected between the two subgroups (*P* = 0.82).

### Lower limb muscle strength: population without disabilities

When comparing the long-term use of KT to minimal intervention, the pooled SMD was significant (SMD = 0.25, 95% CI = 0.01 to 0.50; Fig. 2). Although the result favoured KT, the ES was small. Moderate heterogeneity was detected (I^2^ = 49%). However, when comparing the short-term use of KT to minimal intervention, the pooled SMD was not significant (Fig. 2). As only concentric muscle strength was recorded when both concentric and eccentric strengths were measured by a study, a post hoc sensitivity analysis that separated the analyses of concentric, isometric, and eccentric muscle strengths was conducted (in Additional file 1: Figures S26-S27). Eccentric muscle strength measurements were not excluded in this sensitivity analysis. For short-term KT use, the post hoc sensitivity analyses were not significant. Similarly, the post hoc sensitivity analysis results for long-term KT use for concentric muscle strength was non-significant; however, only two studies were included in this analysis. The result should be interpreted with caution.

### Lower limb muscle strength: chronic musculoskeletal diseases

Two included studies applied KT at the same time of treatment to an antagonistic muscle pair (quadriceps and hamstring) [31, 33]. It is possible that the effects of KT on muscle strength were attenuated by simultaneous activation of both the agonist and antagonist muscles. Therefore, a post hoc subgroup analysis was conducted by dividing the studies into two subgroups: 1) studies in which KT was only applied to the agonist muscle and 2) studies in which KT was applied to an antagonistic muscle pair. The pooled SMD between KT and minimal intervention for the four studies that applied KT to the agonist muscle only was significant (SMD = 1.24, 95% CI = 0.33 to 2.16; Fig. 2). The ES was large and favoured KT. Considerable heterogeneity was detected (I^2^ = 77%). The heterogeneity was due to the study by Anandkumar et al. [30] A post hoc sensitivity analysis was performed after excluding this study (in Additional file 1: Figure S28). The pooled SMD was still significant after the sensitivity analysis (SMD = 0.85, 95% CI = 0.34 to 1.36), and the heterogeneity changed to “not important” (I^2^ = 3%). When comparing KT to minimal intervention, the pooled SMD of the two studies that applied KT to an antagonistic muscle pair was not significant (Fig. 2). The between-subgroup heterogeneity was substantial (I^2^ = 74%). A post hoc sensitivity analysis was conducted by separating analyses for concentric, isometric, and eccentric muscle strengths into different subgroups (in Additional file 1: Figure S29). The pooled SMDs of the subgroups with KT applied only to the agonist muscles measuring isometric and eccentric muscle strengths were not significant in the sensitivity analysis. However, the ES was still positive (favour KT), despite the wider confidence interval due to the decreased number of included studies.

### Lower limb muscle strength: post-operative orthopaedic conditions

In an acute post-operative orthopaedic setting, the pooled SMD between KT and minimal intervention was significant (SMD = 0.60, 95% CI = 0.06 to 1.14; Fig. 2). The ES was moderate and favoured KT. Heterogeneity was not important (I^2^ = 0%). However, only two studies were included, and thus this result should be interpreted with caution. In the non-acute post-operative orthopaedic setting, the pooled SMD between KT and minimal intervention was not significant (Fig. 2). The between-subgroup heterogeneity was considerable (I^2^ = 76.2%). A post hoc sensitivity analysis was conducted by separating analyses for concentric, isometric, and eccentric muscle strengths into different subgroups (in Additional file 1: Figure S30). The resulting interpretation remained the same after sensitivity analysis.

### Hop test: populations with muscle fatigue and post-operative orthopaedic conditions

Only one study was identified for each of the meta-analyses for hop test results in individuals with muscle fatigue and post-operative orthopaedic conditions. There was a significant SMD when comparing the effects of KT to minimal intervention for hop test results in a population with muscle fatigue (SMD = 1.08, 95% CI = 0.31 to 1.86). The ES was large and favoured KT. The SMD between KT and minimal intervention in a population with non-acute post-operative orthopaedic conditions was also significant (SMD = 0.24, 95% CI = 0.12 to 0.36). The ES was small and favoured KT. As only one study was included in the calculation for each of these SMDs, these results should be interpreted with caution. No articles studying patients with chronic musculoskeletal diseases and acute post-operative conditions were identified for this outcome.

### Hop test: populations without disabilities

The meta-analysis results are shown in in the Additional file 1: Figure S1. The SMDs for both short- and long-term KT use versus minimal intervention were not significant in populations without disabilities.

### Vertical jump test: populations with muscle fatigue

Only 2 studies were identified for the short-term effects of KT in populations with muscle fatigue. Similarly, there was only 1 study reporting the long-term effects. For both analyses, the SMD was not significant when comparing KT to a minimal intervention (in Additional file 1: Figure S2 for short-term effect; SMD = 0.35, 95% CI = − 0.32 to 1.03 for long-term effect). These results should be interpreted carefully due to the small number of included studies. There were no identified patients with chronic musculoskeletal diseases or post-operative orthopaedic conditions for this outcome.

### Vertical jump test: populations without disabilities

Only 2 studies investigated the effect of long-term KT use on vertical jump test results in populations without disabilities. There was a significant pooled SMD when comparing long-term KT use to minimal intervention (SMD = 0.18, 95% CI = 0.08 to 0.28; in Additional file 1: Figure S2). Although the result favoured KT, the ES was trivial. Heterogeneity was not important (I^2^ = 0%). As only 2 studies were included, this result should be interpreted with caution. The pooled SMD between short-term KT use and a minimal intervention in subjects without disabilities was not significant (in Additional file 1: Figure S2).

### Publication bias and sensitivity analyses

The details of the publication bias assessments are shown in the Additional file 1: Figures S3-S19. The trim-and-fill method was used to adjust for the identified publication bias. There were no changes in the interpretation of the results after this adjustment.

The details of the planned sensitivity analyses are shown in in the Additional file 1: Figures S20-S22. The exclusion of crossover trials that did not report washout periods and studies that were determined to have poor quality (PEDro score 0–3) produced changes in the interpretation of the results of the following meta-analyses: 1) The pooled SMD between long-term KT use and minimal intervention on lower limb muscle strength in populations without disabilities was no longer significant ( in Additional file 1: Figure S20); 2) The pooled SMD between short-term KT use and minimal intervention for the vertical jump test in populations without disabilities became significant (SMD = 0.11, 95% CI = 0.00 to 0.22; in Additional file 1: Figure S22). However, the ES was trivial; and 3) The pooled SMD between long-term KT use and minimal intervention for the vertical jump test in populations without disabilities was no longer significant (in Additional file 1: Figure S22).

As both parallel and crossover designs were included in this study, post hoc subgroup analyses were conducted by separating these study designs into two subgroups to investigate potential heterogeneity (in Additional file 1: Figure S31-S37). All analyses showed insignificant heterogeneity between the two subgroups (*P* > 0.1). Generally, crossover designs yield narrower confidence intervals than parallel designs. This is because within-subject correlations were taken into account during the calculation of the SMDs, which results in smaller standard errors.

## Discussion

The current meta-analysis establishes evidence for the effectiveness of applying KT to the agonist muscle for improving lower limb muscle strength in individuals with muscle fatigue and chronic musculoskeletal diseases. The ESs for the short-term and long-term effects of KT on lower limb muscle strength in subjects with muscle fatigue were moderate (pooled SMD = 0.53 and 0.61, respectively). Although only two studies were included in the meta-analysis for long-term effects, the results of the meta-analyses favoured KT, which increases the strength of the existing evidence. The ES was large for the meta-analysis of the effect of using KT for lower limb muscle strength in individuals with chronic musculoskeletal diseases (pooled SMD = 1.24). Publication bias analyses did not identify missing studies favouring the control group. This further confirms the beneficial effects of using KT in these populations.

However, there is only weak evidence for the long-term effects of KT on enhancing lower limb muscle strength in populations without disabilities and for KT use in populations with acute post-operative orthopaedic conditions. There was a small ES for long-term KT use in populations without disabilities (pooled SMD = 0.25). The sensitivity analysis yielded an insignificant pooled SMD. These results indicate that long-term KT application may have limited clinical significance in this population. The meta-analysis of 2 studies in populations with acute post-operative conditions yielded significant results with a moderate ES (pooled SMD = 0.60). Nonetheless, this result should be interpreted with caution as the number of studies included in the analysis was small.

There is no evidence that short-term KT use improves lower limb muscle strength or functional performance in populations without disabilities. The present results support the previous findings by Csapo and Alegre [10]. At the same time, the effect of long-term KT application on the hop test results in populations without disabilities was not significant. The evidence for the effect of long-term KT use on vertical jump performance in populations without disabilities is also insufficient. Overall, the findings of this study do not support the use of KT in populations without disabilities.

There is insufficient evidence for the effects of KT on lower limb functional performance in populations with special musculoskeletal conditions (muscle fatigue, chronic musculoskeletal diseases, and post-operative orthopaedic conditions). More randomised controlled trials are required to reach a conclusion in these populations.

### Comparisons with other interventions

Nédélec et al. reported that cold water immersion, compression garments, and massage are commonly used for muscle fatigue recovery after exercise [59]. Among the meta-analyses investigating the effectiveness of these interventions on strength recovery in fatigued muscle [60–62], compression garments produced the largest ES compared to the control group (pooled SMD = 0.462, 95% CI = 0.221 to 0.703) [61]. In the current study, KT yielded a larger ES (pooled SMD = 0.53 and 0.61 for short-term and long-term effects, respectively) for lower limb muscle strength than the abovementioned therapies. This result indicates that KT may be a better intervention for muscle fatigue recovery. Resistance exercises and neuromuscular electrical stimulation (NMES) are the most common physical modalities for muscle strength enhancement in populations with chronic musculoskeletal diseases [63]. Bartholdy et al. indicated that muscle strength training following the American College of Sports Medicine guidelines is more effective than a control for increasing knee extensor strength in patients with knee osteoarthritis (pooled SMD = 0.83, 95% CI = 0.49 to 1.17) [64]. A meta-analysis by Giggins et al. reported that there is inconsistent evidence for the effect of NMES on improving strength in the quadriceps muscle in individuals with knee osteoarthritis [65]. In this study, KT yielded a larger ES (pooled SMD = 1.24) for lower limb muscle strength than the abovementioned modalities. In addition, Lim and Tay reported that KT is more effective than minimal intervention (sham or no taping) for treating chronic musculoskeletal pain [9]. Therefore, KT may be an effective treatment for chronic musculoskeletal diseases, producing both pain relief and increased muscle strength.

### Underlying mechanisms of KT

To date, the underlying mechanisms of KT have not been thoroughly investigated. However, there are several postulated mechanisms. First, KT may increase local blood circulation at the application site. Windisch et al. concluded that KT was better than the A-V Impulse Foot Compression System in improving local blood circulation in the knee over a 7-day application period after total knee arthroplasty (TKA) [66]. Aguilar-Ferrándiz et al. reported that using KT for one month could improve the venous refill time and venous pump function compared to using placebo taping in females with post-menopausal chronic venous insufficiency [67]. In contrast, Yang and Lee reported that KT did not increase local blood circulation in the lower back over a 15-min period in a population without disabilities [68]. Woodward et al. indicated that 30 min of KT use was not superior to no tape for improving blood flow in the skin in a population without disabilities [69]. These conflicting results may be explained by the differences in application period and population characteristics between these studies. Studies by Windisch et al. and Agular-Ferrándiz et al. had longer KT application periods (7 days and 1 month, respectively), and the study populations had musculoskeletal and circulation impairments (post-TKA and chronic venous insufficiency, respectively). Therefore, KT may still be effective for patients with impairments but not for those without disabilities. For the studies conducted in populations with muscle fatigue, the fatigue statuses were induced by isometric [11, 15], concentric [13], eccentric [12, 14], and reciprocal concentric/eccentric exercises [39]. It is known that eccentric and isometric exercises can induce muscle damage and soreness [70]. Therefore, KT may be beneficial to populations with muscle fatigue and chronic musculoskeletal diseases as increased blood circulation facilitates recovery by increasing nutrient and waste exchange. Second, KT may suppress pain via the mechanism proposed by the gate control theory [71]. KT is able to provide tactile stimulation [72]. This stimulation may lead to the firing of large-diameter afferent fibres, which close the gate to pain signals transmitted by small-diameter afferent fibres. This stimulation then produces a decrease in muscle soreness and musculoskeletal pain and enhances muscle strength. Some meta-analyses have reported evidence for the pain-relieving effect of KT [6, 7, 9]. These two postulated mechanisms explain why KT is effective in the populations with muscle fatigue and chronic musculoskeletal diseases, but not in the population without disabilities. Third, KT may increase muscle strength by alternating fascia movements. Tu et al. reported that KT changed the motion of fascia during trunk flexion [73]. Findley et al. reported that substantial force in the muscle was transmitted to the fascia [74]. Therefore, KT may facilitate muscle strengthening by transmitting a pulling force to the muscle and fascia, as mentioned by Kuo and Huang [3]. Fourth, Yeung and Yeung proposed that KT may stimulate skin mechanoreceptors [4]. If the direction in which KT is pulling matches the direction of the muscle contraction, then KT could enhance the muscle spindle reflex and increase the excitability of the motor units; if the directions of the pulling force and muscle contraction are in opposite directions, then KT can stretch the Golgi tendon organs and reduce the activity of the corresponding motor neuron. Currently, there is insufficient research examining the physiological mechanisms of the facilitatory and inhibitory properties of KT in muscle contractions. More physiological studies are thus warranted.

### Clinical implications

The current findings suggest that KT may be an ergogenic agent for recovering from lower limb muscle fatigue. Unlike other common interventions for fatigue recovery, including cold water immersion, KT can be kept on the skin while participating in sports activities and can provide continuous treatment for the muscles. KT may also be an effective modality for increasing lower limb muscle strength in populations with chronic musculoskeletal diseases. Segal et al. reported that high knee extensor strength protects against symptom development in knee osteoarthritis [75]. However, KT should not be applied on an antagonistic muscle pair. It is plausible that KT application on antagonist muscles can stimulate antagonist muscles during voluntary agonist contractions. This results in the reciprocal inhibition of the agonist contraction.

### Strengths

First, this study used a different approach than the previous meta-analysis that reported non-significant results for the effectiveness of KT [10]. The present study is the first meta-analysis of randomised controlled trials that focused specifically on the effectiveness of facilitatory KT. The results favoured using KT for improving lower limb muscle strength in individuals with chronic musculoskeletal diseases and muscle fatigue. Second, this study included different population groups to guide clinical judgements. Third, this study included muscle strength and functional performance testing as outcomes to provide more evidence for clinical practice.

### Limitations

First, this study included a relatively small number of studies in the meta-analyses of the functional performance tests. Second, this study did not investigate the effectiveness of KT on upper limb function. However, this approach was used to avoid potential heterogeneity related to the physiological differences between the upper and lower extremities. Third, this study did not assess the effect of KT in other population groups (e.g., individuals with neurological diseases). This study focused on musculoskeletal conditions to provide specific clinical evidence. Fourth, this study did not evaluate other functional performance tests (e.g., sprint speed). Fifth, after conducting the literature search, several papers with good study designs were identified. However, those studies were not included because they did not meet the eligibility criteria. Aktas and Baltaci measured the effect of KT on knee muscle strength and distance in the single-leg hop and vertical jump tests [52]. However, the standard deviation of the results was not provided in the study by Aktas and Baltaci. In addition, Bicici et al. measured the effect of KT on different functional performance measurements, including hopping, vertical jump, and dynamic balance [40]. However, the study used an inhibitory KT application, which did not fall within the inclusion criteria of the present study. Sixth, because multiple brands and types of KT are used among the randomised-controlled trials, this study did not require a specific brand or type of KT in the eligibility criterion. This decision helped to avoid a substantial reduction in sample size. However, this may have produced some potential clinical heterogeneity owing to differences between different types of KT.

### Future research

Further study is warranted to investigate the effect of facilitatory KT on different types of athletic performance (e.g., agility tests, sprint speed, ball speed) in individuals with muscle fatigue and various lower limb functions (e.g., sit-to-stand, stair walking) in individuals with chronic musculoskeletal diseases. Studies ascertaining the mechanisms of KT are also needed.

## Conclusion

The current study concluded that facilitatory KT use is effective for improving lower limb muscle strength in populations with muscle fatigue and chronic musculoskeletal diseases compared to minimal interventions. It is recommended that KT be applied only to agonist muscles. The results of the present study do not support KT use in populations without disabilities. There is insufficient evidence for the effects of KT on functional performance in populations with special musculoskeletal conditions (muscle fatigue, chronic musculoskeletal diseases, and post-operative orthopaedic conditions).

## Additional files

Additional file 1: Appendix 1) Meta-analyses for functional performance tests 2) Publication bias analyses 3) Sensitivity and subgroup analyses 4) Characteristics of included studies 5) PEDro scale scoring 6) Search strategies (DOCX 622 kb)

Additional file 2: PRISMA 2009 checklist. A completed PRISMA checklist (DOC 66 kb)

## Abbreviations

ES: Effect size
KT: Kinesio Taping
NMES: Neuromuscular electrical stimulation
SD: Standard deviation
SE: Standard error
SMD: Standardized mean difference
TKA: Total knee arthroplasty
WD: Within-participant difference
