# Supplementary material for: Effects of Kinesio tape on lower limb muscle strength, hop test, and vertical jump performances: a meta-analysis
Source: BMC Musculoskelet Disord. 2019 May 14;20:212. doi: 10.1186/s12891-019-2564-6 (PMC6518687; doi:10.1186/s12891-019-2564-6)
Supplement: Supplementary file 1 — Appendix 1) Meta-analyses for functional performance tests 2) Publication bias analyses 3) Sensitivity and subgroup analyses 4) Characteristics of included studies 5) PEDro scale scoring 6) Search strategies (DOCX 622 kb) [file 12891_2019_2564_MOESM1_ESM.docx]

**Additional file 1**


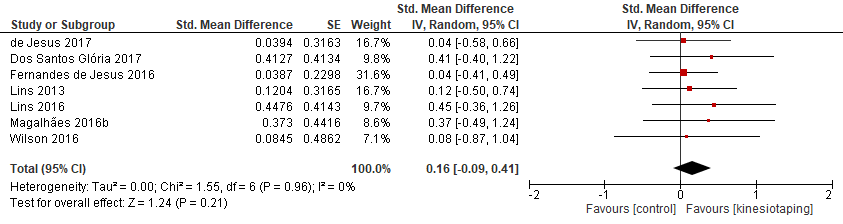


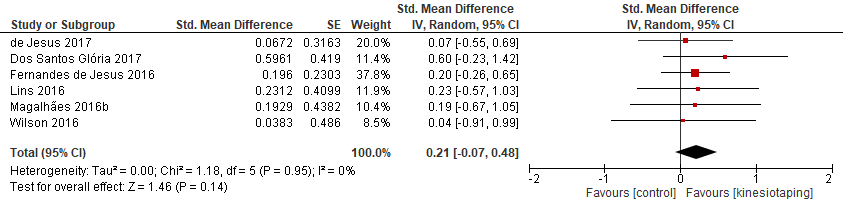


**Figure S1** Meta-analyses for the effect of KT on distance in a single-leg hop

- The order of population groups from the top to the bottom (Short-term KT application in population without disabilities, and long-term KT application in population without disabilities)


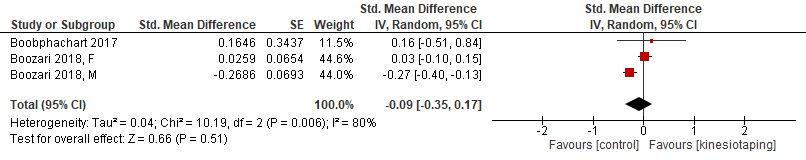


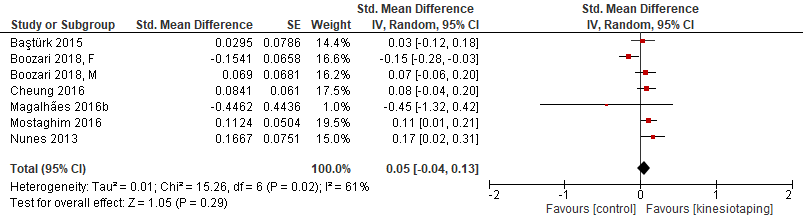


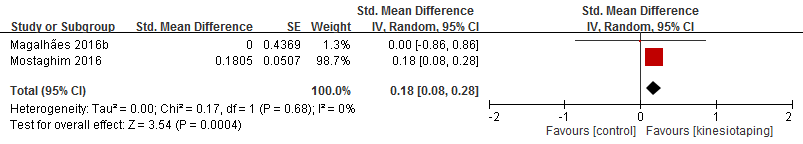


**Figure S2** Meta-analyses for the effect of KT on vertical jump height

- The order of population groups from the top to the bottom (Short-term effect of KT application in population with muscle fatigue, short-term KT application in population without disabilities, and long-term KT application in population without disabilities)


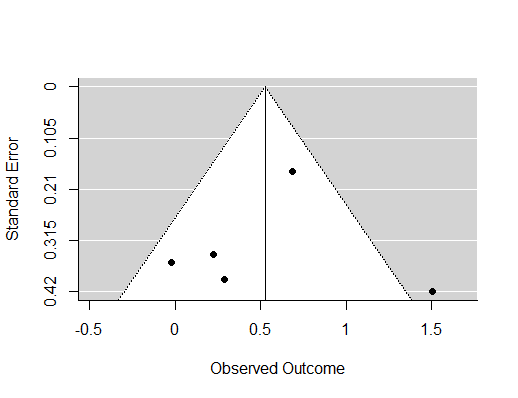


**Figure S3** Funnel plot for the short-term effect of KT application on lower limb muscle strength in population with muscle fatigue

- By visual inspection, there was a gap in the bottom right corner of the graph.
- By trim-and-fill method, the estimated number of missing studies on the left side was 0. The estimated number of missing studies on the right side was 1.


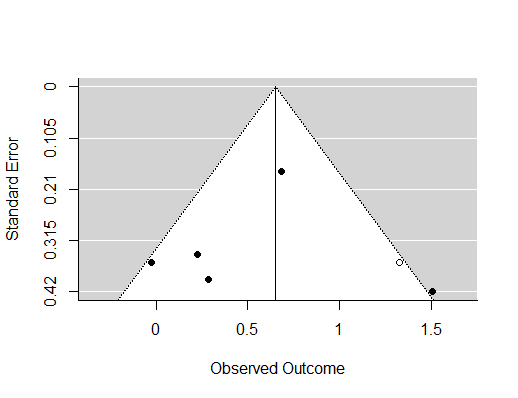


**Figure S4** Funnel plot for the short-term effect of KT application on lower limb muscle strength in population with muscle fatigue after adjusting for missing studies at the right side by trim-and-fill method

- SMD = 0.65, 95%CI = 0.22 to 1.08
- There was no change in result interpretation.


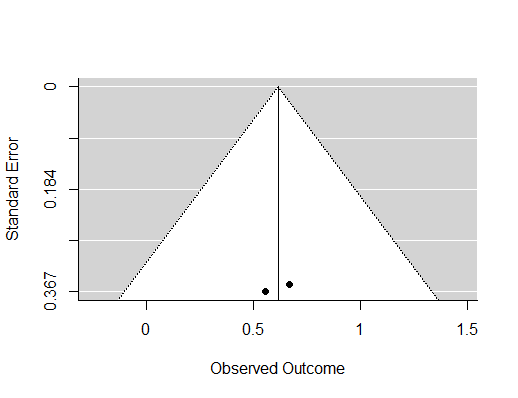


**Figure S5** Funnel plot for the long-term effect of KT application on lower limb muscle strength in population with muscle fatigue

- Publication bias could not be estimated owing to only two included studies.


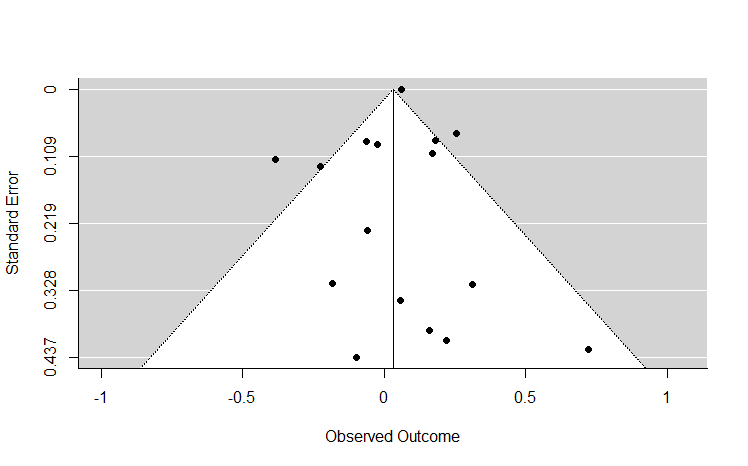


**Figure S6** Funnel plot for the effect of short-term application of KT on lower limb muscle strength in population without disabilities

- Egger’s test result: Z = 0.3671, p = 0.7136
- No significant publication bias was detected.


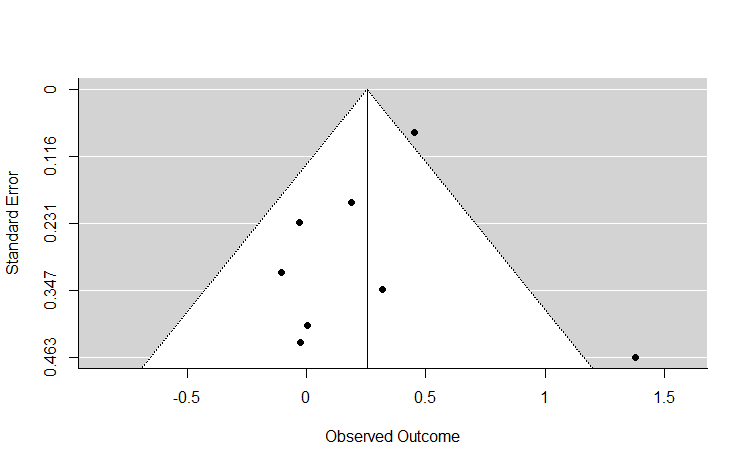


**Figure S7** Funnel plot for the effect of long-term application of KT on lower limb muscle strength in population without disabilities

- By visual inspection, there was a gap in the bottom right corner of the graph.
- By trim-and-fill method, the estimated number of missing studies on the left side was 0. The estimated number of missing studies on the right side was 2.


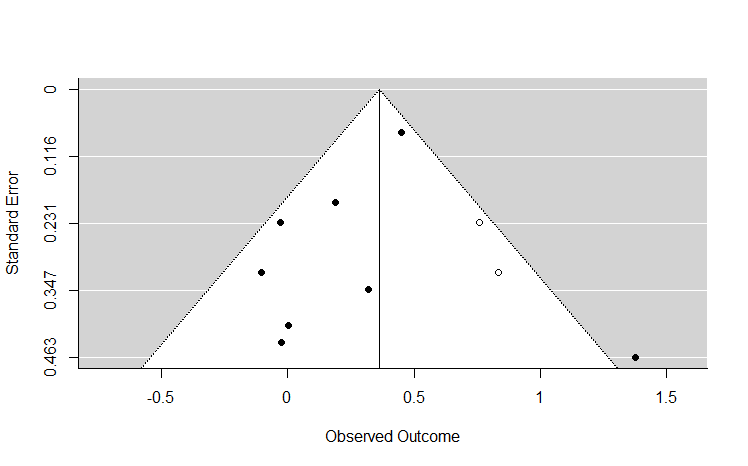


**Figure S8** Funnel plot for the effect of long-term application of KT on lower limb muscle strength in population without disabilities after adjusting for missing studies at the right side by trim-and-fill method

- SMD = 0.36, 95%CI = 0.14 to 0.59
- There was no change in result interpretation.


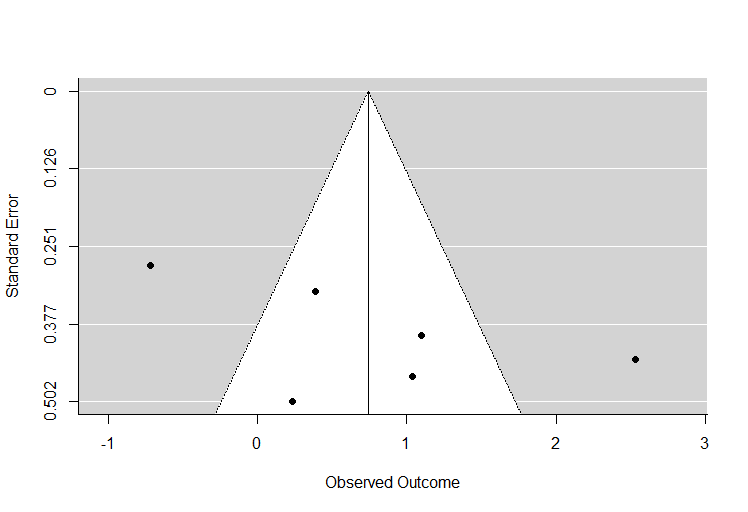


**
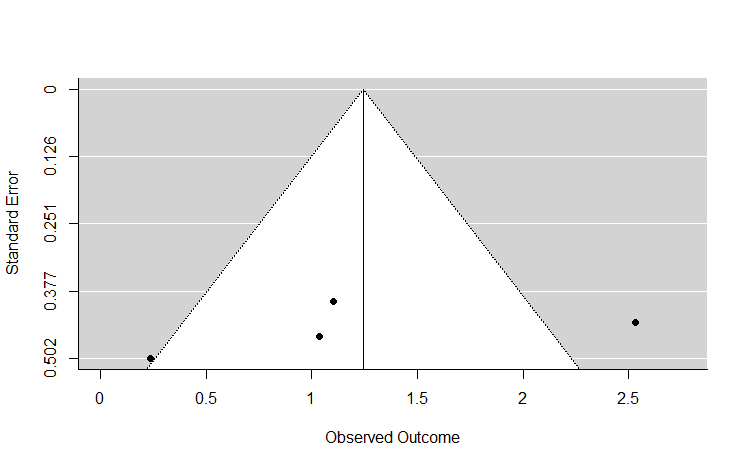

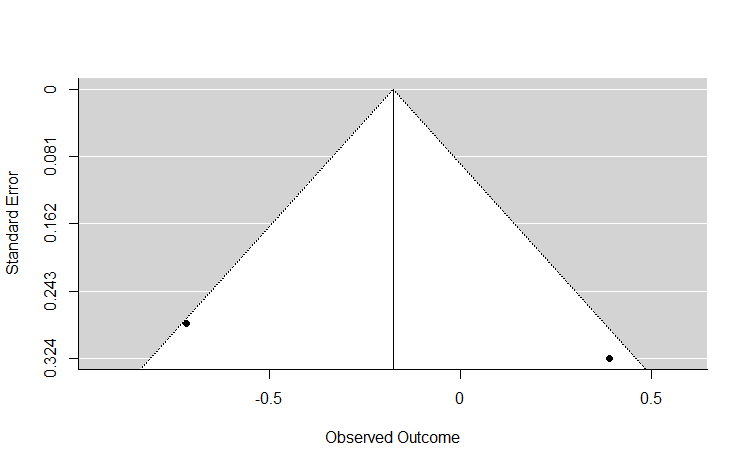
**

**Figure S9** Funnel plots for the effect of KT on lower limb muscle strength in population with chronic musculoskeletal diseases

- The order of funnel plots from the top to the bottom (All studies, subgroup with KT applied on agonist only, and subgroup with KT applied on antagonist muscle pair)
- By visual inspection, the funnel plot was symmetrical for the funnel plots of all studies. There was a gap in the bottom right corner of the funnel plot of subgroup with KT applied on agonist only. Symmetry could not be assessed for subgroup with KT applied on antagonist muscle pair as only two studies were included.
- By trim-and-fill method, for the funnel plot of all studies, the estimated number of missing studies on the left side was 0. The estimated number of missing studies on the right side was 0. For the funnel plot of subgroup with KT applied on agonist only, the estimated number of missing studies on the left side was 0. The estimated number of missing studies on the right side was 1. Trim-and-fill method could not be conducted for the subgroup with KT applied on antagonist muscle pair.


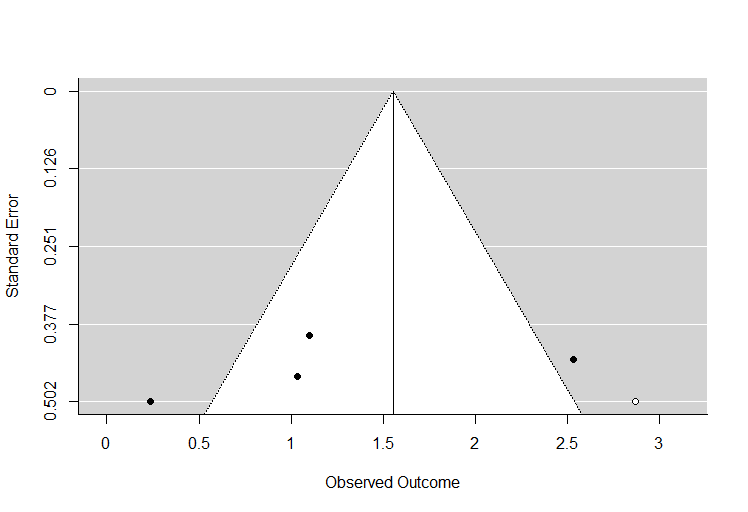


**Figure S10** Funnel plot for the effect of KT on lower limb muscle strength in population with chronic musculoskeletal diseases after adjusting for missing studies at the right side by trim-and-fill method

- SMD = 1.55, 95%CI = 0.63 to 2.48
- There was no change in result interpretation.

**
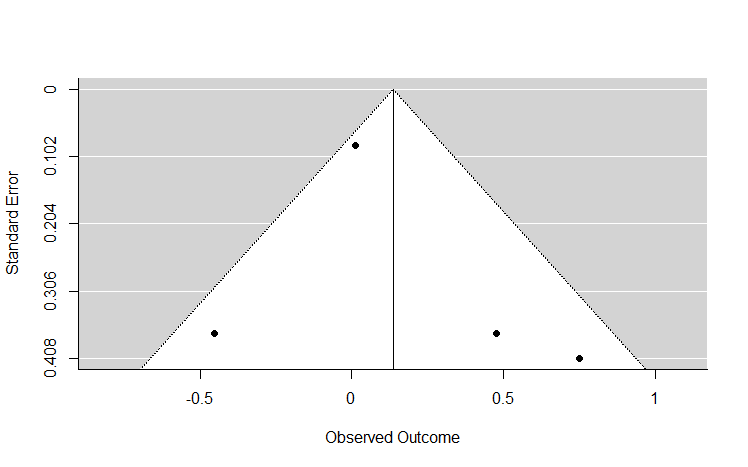
**


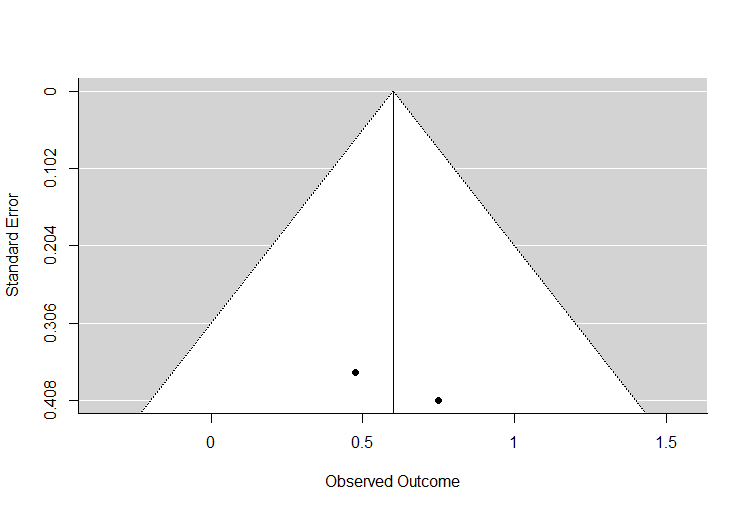


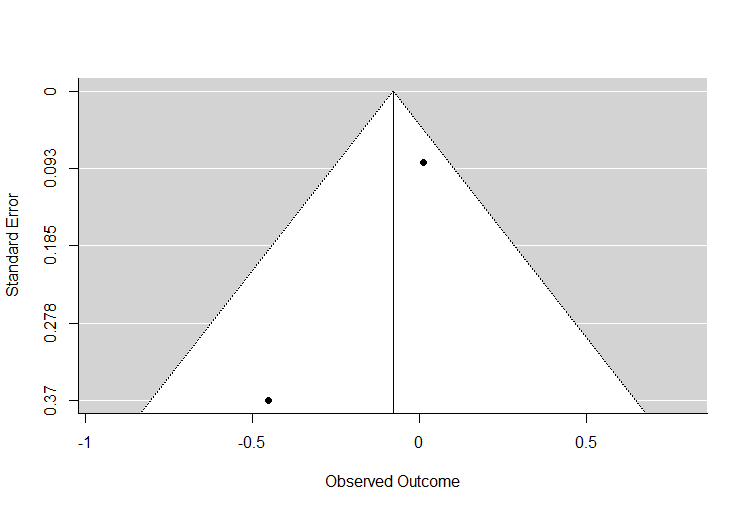


**Figure S11** Funnel plot for the effect of KT on lower limb muscle strength in population under post-operative orthopaedic conditions

- The order of funnel plots from the top to the bottom (All studies, subgroup of acute setting, and subgroup of non-acute setting)
- By visual inspection, there was a gap in the bottom left corner of the funnel plot of all studies. Symmetry could not be assessed for the subgroups owing to small number of studies.
- By trim-and-fill method, for the funnel plot of all studies, the estimated number of missing studies on the left side was 1. The estimated number of missing studies on the right side was 0. Trim-and-fill method could not be conducted for the subgroups.


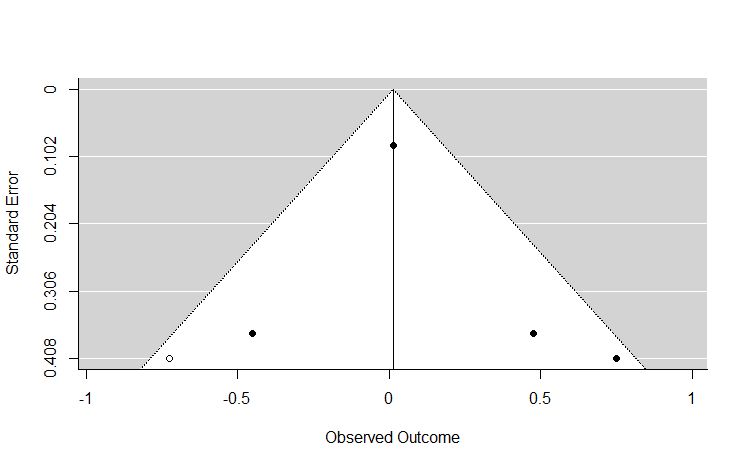


**Figure S12** Funnel plot for the effect of KT on lower limb muscle strength in population under post-operative orthopaedic conditions after adjusting for missing studies at the left side by trim-and-fill method

- SMD = 0.01, 95%CI = -0.40 to 0.42
- There was no change in result interpretation.

**
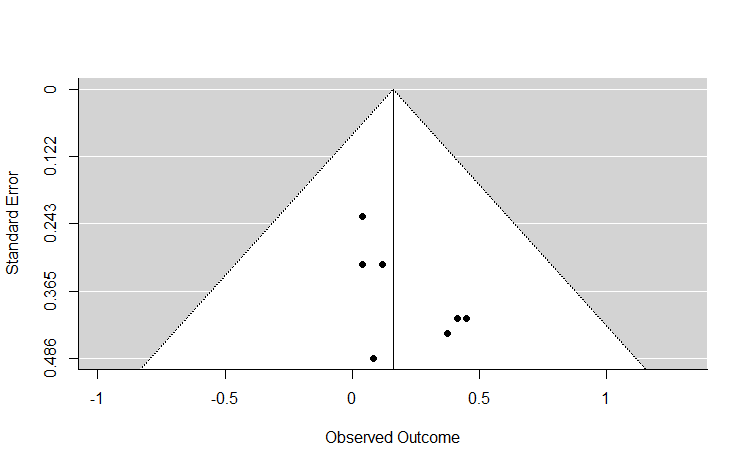
**

**Figure S13** Funnel plot for the effect of short-term application of KT on hop test result in population without disabilities

- By visual inspection, there was a gap in the bottom left corner of the graph.
- By trim-and-fill method, the estimated number of missing studies on the left side was 1. The estimated number of missing studies on the right side was 0.


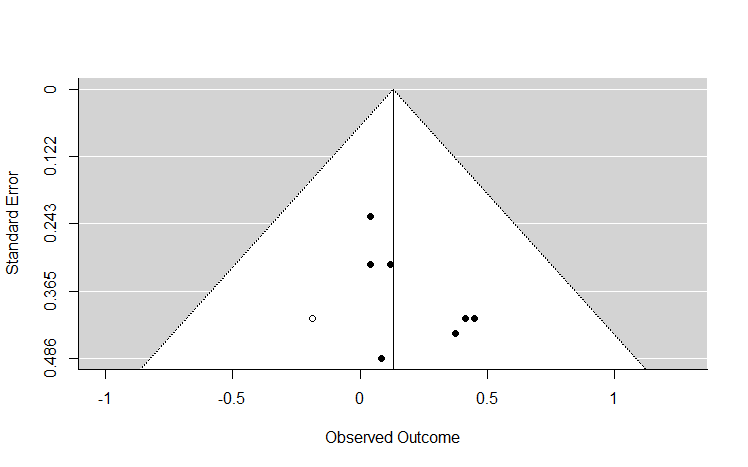


**Figure S14** Funnel plot for the effect of short-term application of KT on hop test result in population without disabilities after adjusting for missing studies at the left side by trim-and-fill method

- SMD = 0.13, 95%CI = -0.11 to 0.37
- There was no change in result interpretation.

**
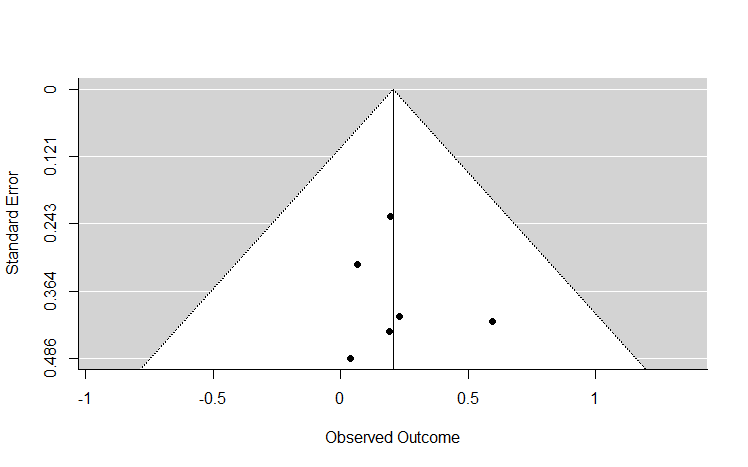
**

**Figure S15** Funnel plot for the effect of long-term application of KT on hop test result in population without disabilities

- By visual inspection, there was a gap in the bottom right corner of the graph.
- By trim-and-fill method, the estimated number of missing studies on the left side was 0. The estimated number of missing studies on the right side was 1.


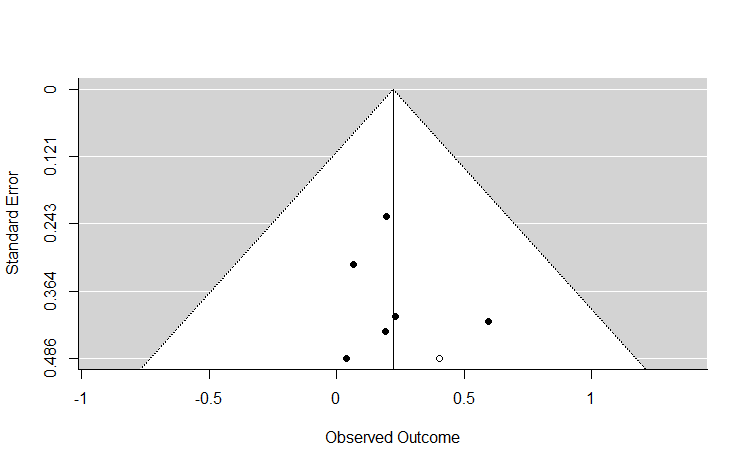


**Figure S16** Funnel plot for the effect of long-term application of KT on hop test result in population without disabilities after adjusting for missing studies at the right side by trim-and-fill method

- SMD = 0.22, 95%CI = -0.04 to 0.49
- There was no change in result interpretation.


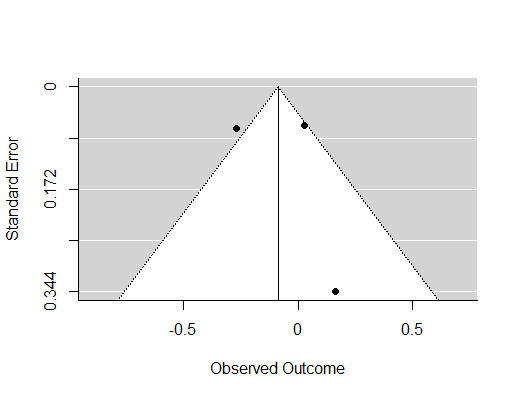


**Figure S17** Funnel plot for the short-term effect of KT application on vertical jump test result in population with muscle fatigue

- Publication bias could not be estimated owing to only two included studies (the two points in the top left corner were from male and female subjects of the same study).


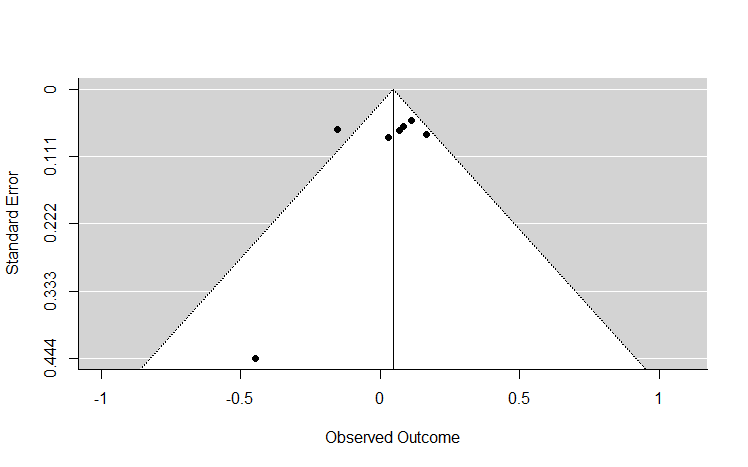


**Figure S18** Funnel plot for the effect of short-term application of KT on vertical jump test result in population without disabilities

- By visual inspection, there was a gap in the bottom right corner of the graph.
- By trim-and-fill method, the estimated number of missing studies on the left side was 0. The estimated number of missing studies on the right side was 0.
- Trim-and-fill method could not be implemented owing to no estimated missing study.


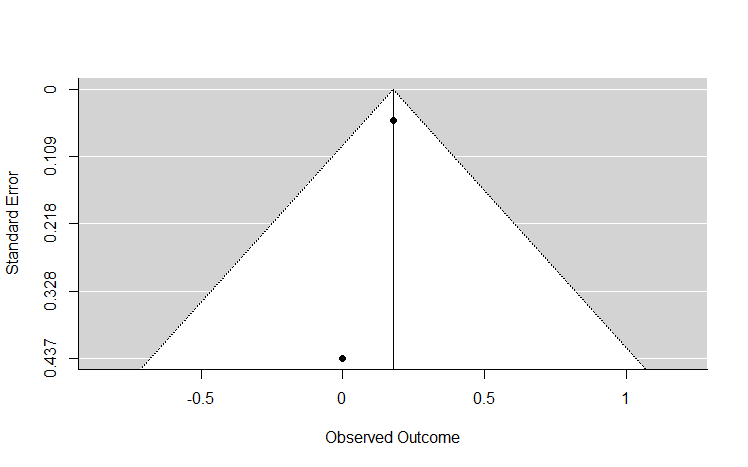


**Figure S19** Funnel plot for the effect of long-term application of KT on vertical jump test result in population without disabilities

- Publication bias could not be estimated owing to only two included studies.


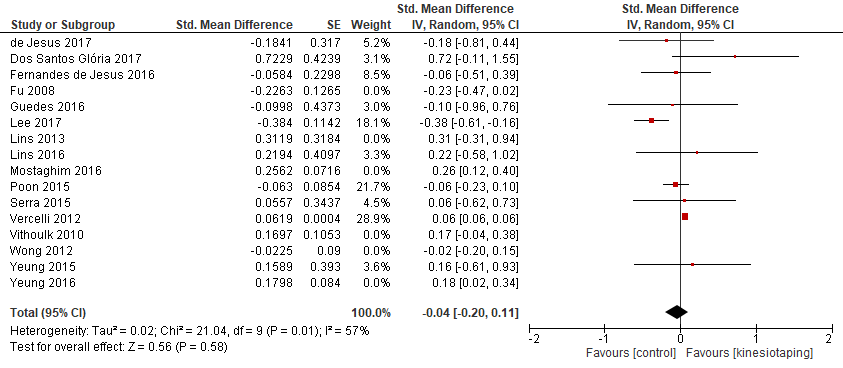


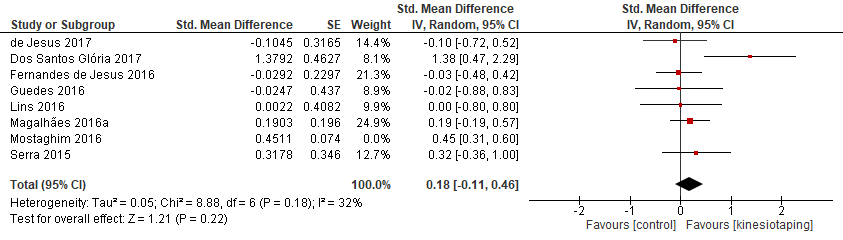


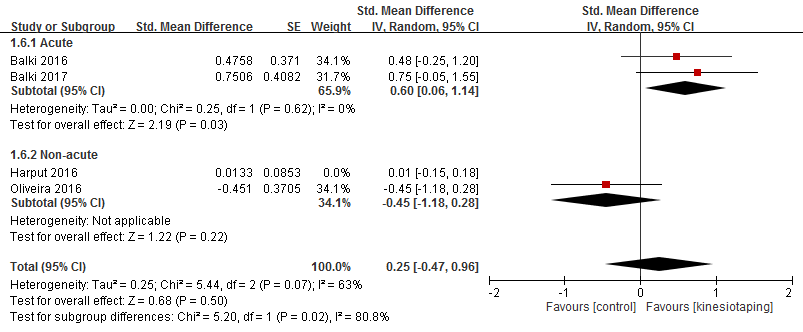


**Figure S20** Planned sensitivity analyses on lower limb muscle strength

- The order of population group from the top to the bottom (Short-term KT application on population without disabilities, long-term KT application on population without disabilities, and population under post-operative orthopaedic conditions)


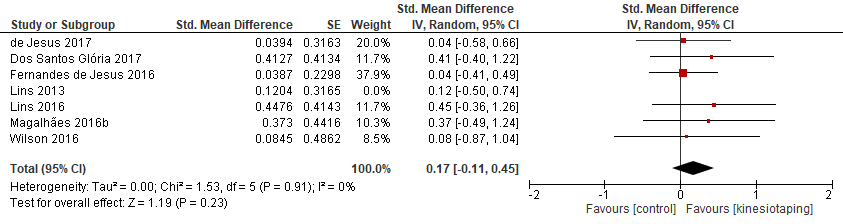


**Figure S21** Planned sensitivity analysis on distance in a single-leg hop

- Population group (Short-term KT application on population without disabilities)


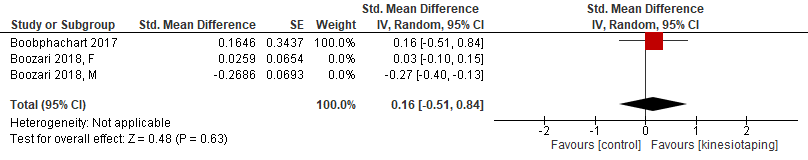


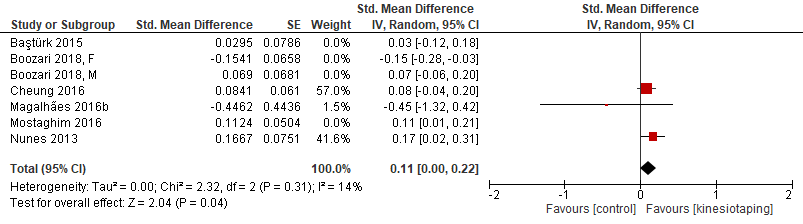


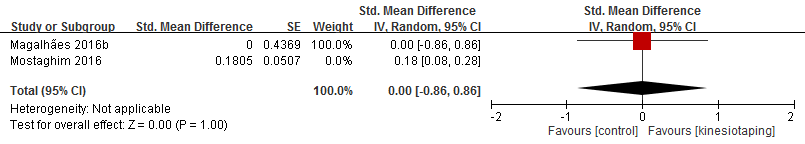


**Figure S22** Planned sensitivity analyses on vertical jump test result

- The order of population group from the top to the bottom (Short-term effect of KT application in population with muscle fatigue, short-term KT application in population without disabilities, and long-term KT application in population without disabilities)


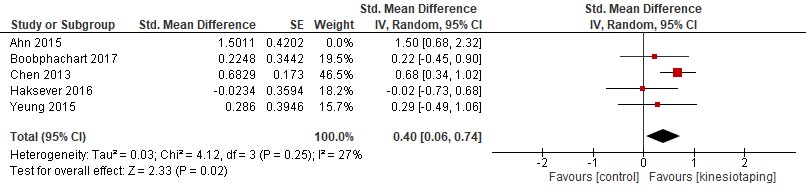


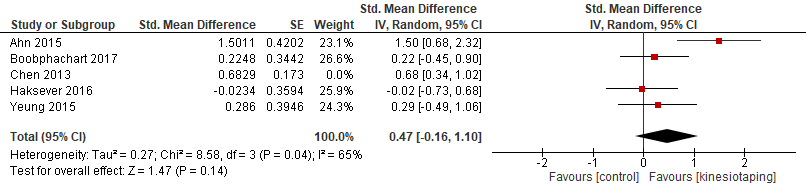


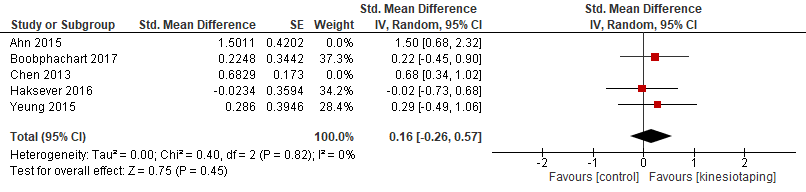


**Figure S23** Post-hoc sensitivity analyses for short-term effect of KT application on lower limb muscle strength in population with muscle fatigue

- The first figure displays the sensitivity analysis by excluding a study causing substantial heterogeneity.
- The second figure displays the sensitivity analysis by excluding a study using “add-on” design with active treatment as control.
- The third figure displays the sensitivity analysis by excluding the above two studies.


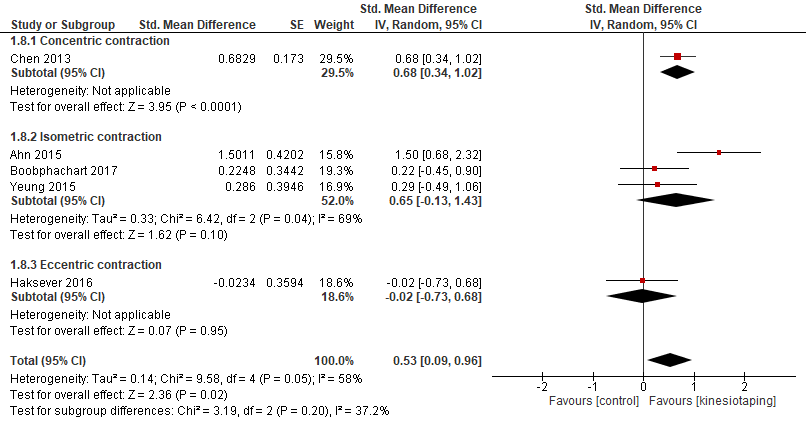


**Figure S24** Post-hoc subgroup analyses for short-term effect of KT application on lower limb muscle strength in population with muscle fatigue

- Separate studies measuring concentric, isometric, and eccentric contractions


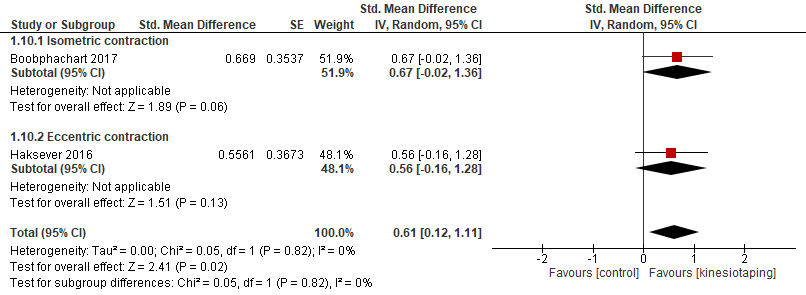


**Figure A25** Post-hoc subgroup analyses for long-term effect of KT application on lower limb muscle strength in population with muscle fatigue

- Separate studies measuring isometric, and eccentric contractions


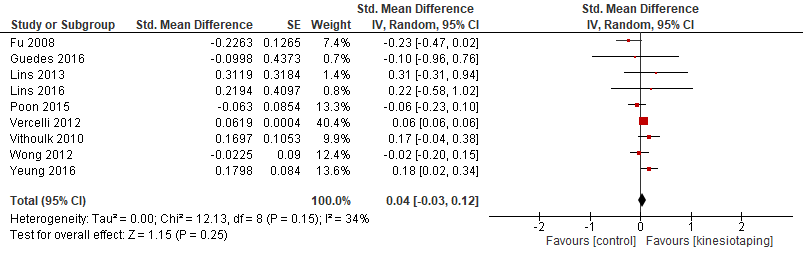


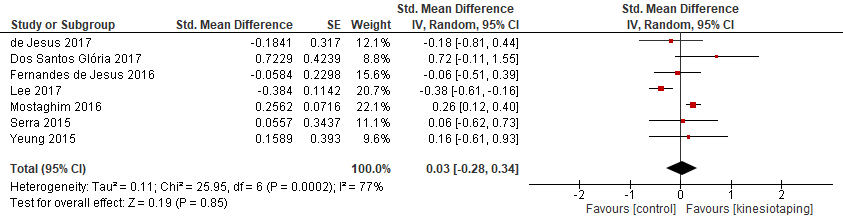


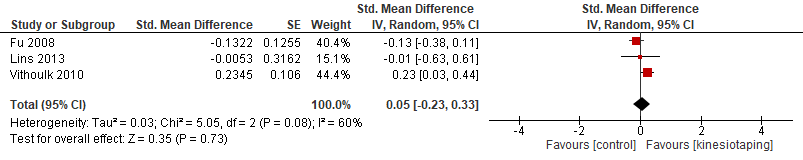


**Figure S26** Post-hoc sensitivity analyses for the effect of short-term application of KT on lower limb muscle strength in population without disabilities

- Separate the analyses for concentric, isometric, and eccentric muscle strengths
- The order of outcome from the top to the bottom (Concentric, isometric, and eccentric contraction)


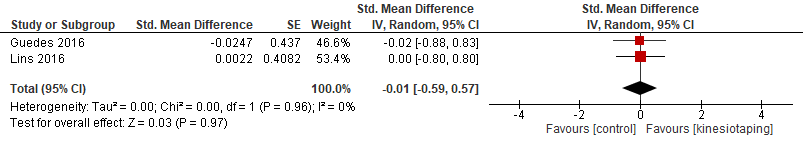


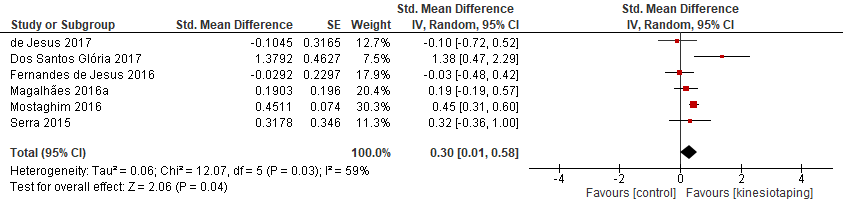


**Figure S27** Post-hoc sensitivity analyses for the effect of long-term application of KT on lower limb muscle strength in population without disabilities

- Separate the analyses for concentric and isometric muscle strengths
- The order of outcome from the top to the bottom (Concentric and isometric contraction)


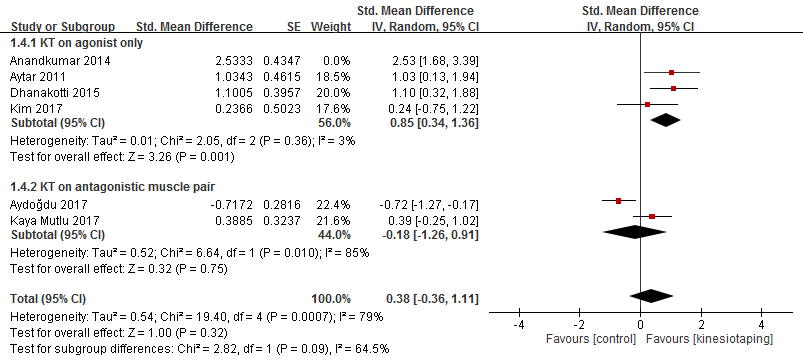


**Figure S28** Post-hoc sensitivity analysis on lower limb muscle strength in population with chronic musculoskeletal diseases

- The sensitivity analysis was performed by excluding a study causing considerable heterogeneity.


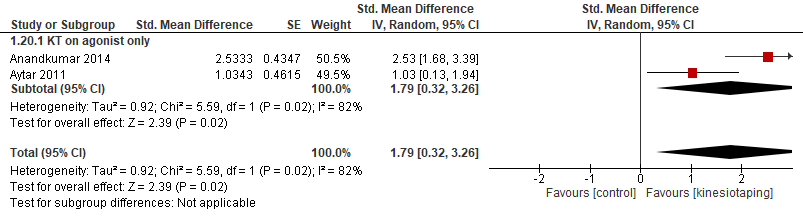


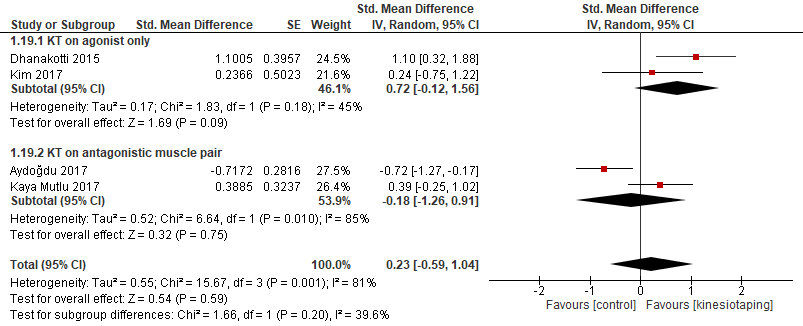


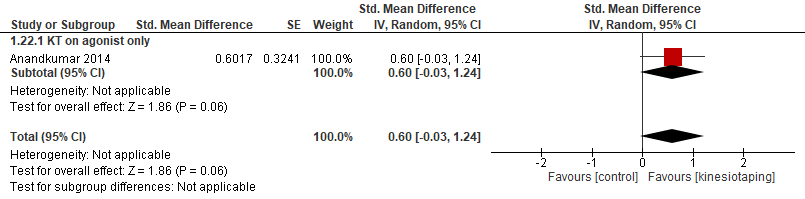


**Figure S29** Post-hoc sensitivity analyses for the effect of KT on lower limb muscle strength in population with chronic musculoskeletal diseases

- Separate the analyses for concentric, isometric, and eccentric muscle strengths
- The order of outcome from the top to the bottom (Concentric, isometric, and eccentric contraction)


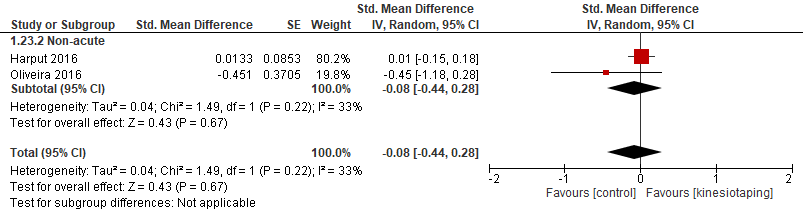


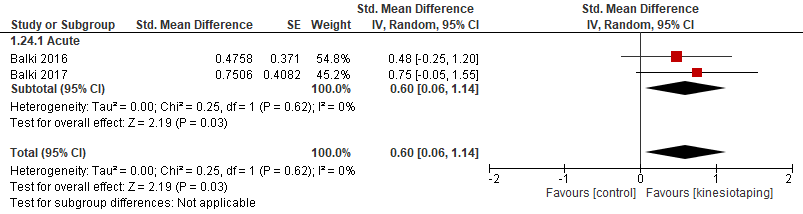


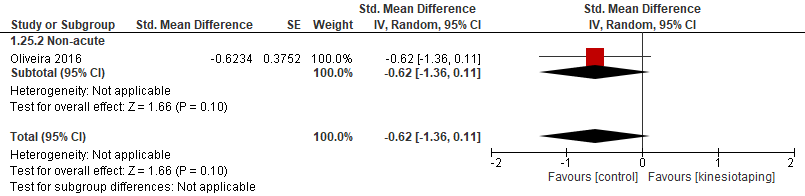


**Figure S30** Post-hoc sensitivity analyses for the effect of KT on lower limb muscle strength in population under post-operative orthopaedic conditions

- Separate the analyses for concentric, isometric, and eccentric muscle strengths
- The order of outcome from the top to the bottom (Concentric, isometric, and eccentric contraction)


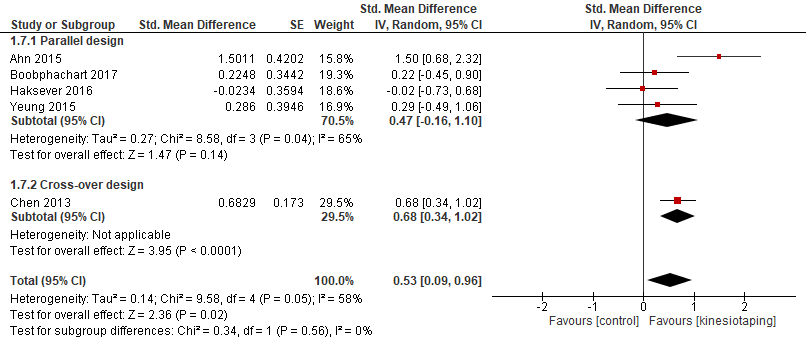


**Figure S31** Post-hoc subgroup analysis for short-term effect of KT application on lower limb muscle strength in population with muscle fatigue

- Separate the analyses for parallel and cross-over design


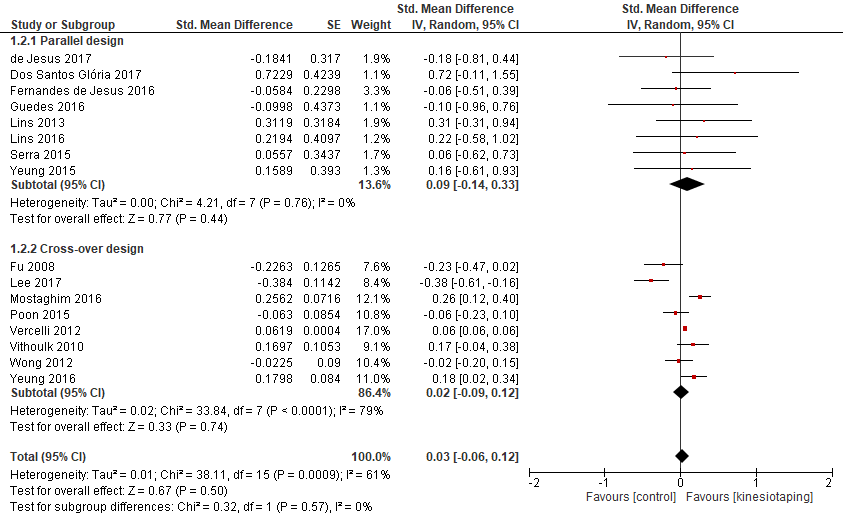


**Figure S32** Post-hoc subgroup analysis for short-term effect of KT application on lower limb muscle strength in population without disability

- Separate the analyses for parallel and cross-over design


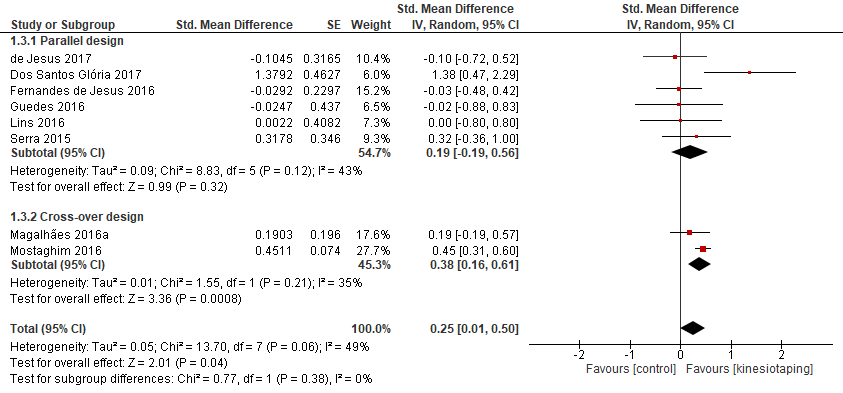


**Figure S33** Post-hoc subgroup analysis for long-term effect of KT application on lower limb muscle strength in population without disability

- Separate the analyses for parallel and cross-over design


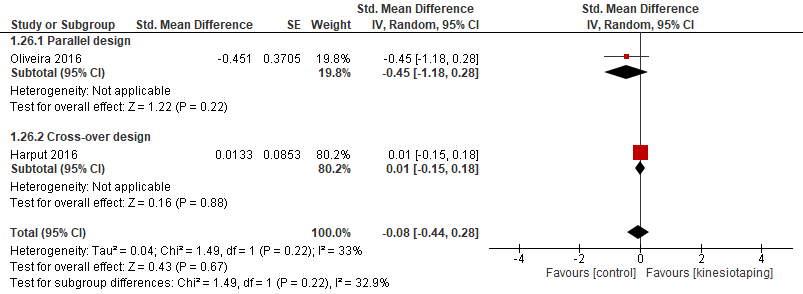


**Figure S34** Post-hoc subgroup analysis for the effect of KT application on lower limb muscle strength in population under non-acute post-operative orthopaedic condition

- Separate the analyses for parallel and cross-over design


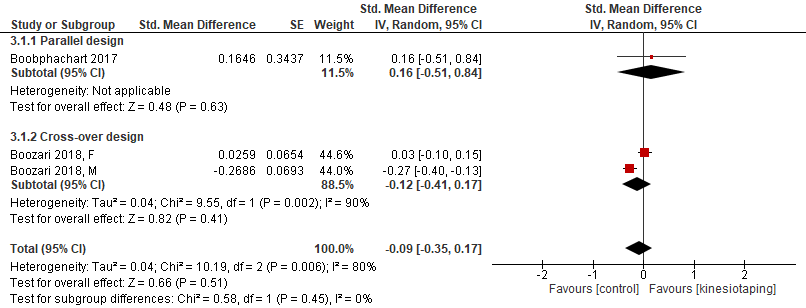


**Figure S35** Post-hoc subgroup analysis for the effect of KT application on vertical jump test result in population with muscle fatigue

- Separate the analyses for parallel and cross-over design


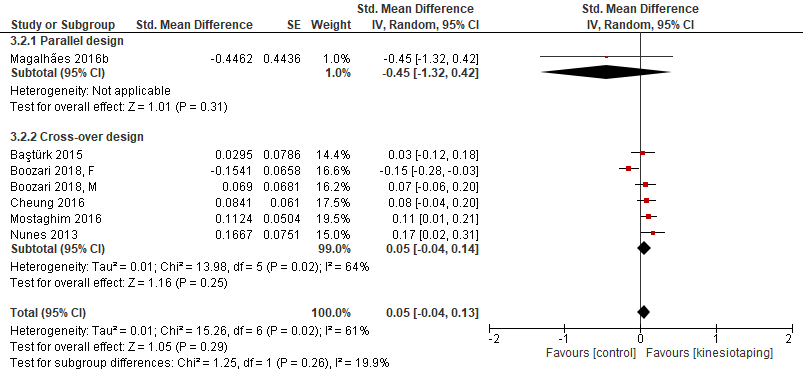


**Figure S36** Post-hoc subgroup analysis for the short-term effect of KT application on vertical jump test result in population without disability

- Separate the analyses for parallel and cross-over design


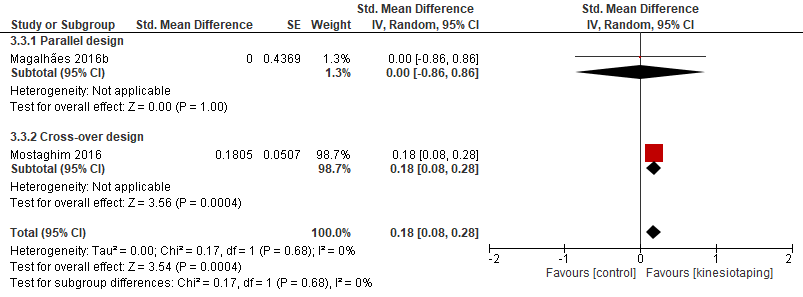


**Figure S37** Post-hoc subgroup analysis for the long-term effect of KT application on vertical jump test result in population without disability

- Separate the analyses for parallel and cross-over design

| **Author, Year (Fatigue protocol)** | **Study design (washout period)** | **Intervention (N)** | **Age [year(SD)]** | **Gender (M, F)** | **Brief details of intervention** | **Outcome measures included** |
| --- | --- | --- | --- | --- | --- | --- |
| Ahn, 2015^11^ (Isometric exercise) | RCTP | KT (15) PT (15) NT (15) | KT 29.06(2.84) PT 31.33(4.11) NT 30.26(3.41) | KT (0, 15) PT (0, 15) NT (0, 15) | KT: Facilitation, 3 strips (RF, VL, VM), 40% tension, Y-shaped PT: Transverse, 2 strips, no tension, I-shaped NT: No tape | Isometric knee extensor strength Distance in a single-leg hop |
| Boobphachart, 2017^12^ (Eccentric exercise) | RCTP | KT (17) PT (17) SS (17) | KT 43.9(1.4) PT 43.1(2.0) SS 40.3(2.2) | KT (0, 17) PT (0, 17) SS (0, 17) | KT: Facilitation, 3 strips (RF, VL, VM), 125% length, Y-shaped (RF), I-shaped (VL, VM) PT: 3 strips (RF, VL, VM), no tension, I-shaped SS: Static stretching | Isometric knee extension peak torque Counter-movement jump height |
| Boozari, 2018^13^ (Concentric exercise) | RCTC (Not indicated) | KT (50) NT (50) | M 26.62(4.45) F 28.15(3.67) | 24, 26 | KT: Facilitation, 1 strip (GN), 35% tension, Y-shaped NT: No tape | Single-leg countermovement jump height |
| Chen,2013^39^ (Reciprocal concentric/eccentric exercise) | RCTC (7 days) | KT+ SS (9) PNF + SS (9)  NT (9) | 23.9(3.1) | 9, 0 | KT + SS: Facilitation, 2 strips (HS), 120% length, V-shaped + Static stretching PNF + SS: PNF stretching + Static stretching NT: No tape | Isokinetic concentric knee flexion peak torque (180°/s) |

**Table S1** Characteristics of the included studies in population with muscle fatigue

Continued

**Table S1** Continued

| Author, Year (Fatigue protocol) | Study design (washout period) | Intervention (N) | Age [year(SD)] | Gender (M, F) | Brief details of intervention | Outcome measures included |
| --- | --- | --- | --- | --- | --- | --- |
| Haksever, 2016^14^ (Eccentric exercise) | RCTP | KT (16) NT (15) | KT 23.2(1.5) NT 23.9(1.0) | KT (0, 16) NT (0, 15) | KT: Facilitation, 4 strips (RF, VMO, BF, ST & SM), 10-30% tension (RF, VMO), 30% tension (BF, ST & SM), Y-shaped NT: No tape | Isokinetic eccentric knee extension peak torque (30°/s) |
| Yeung, 2015^15^ (Isometric exercise) | RCTP | KT (13) PT (13) | KT 22.62(2.93) PT 23.31(3.97) | KT (5, 8) PT (8, 5) | KT: Facilitation, 1 strip (VMO), 50% tension, Y-shaped PT: Non-stretchable Fixomull tape, same application as KT | Isometric knee extension peak torque |

RCTP: Randomized Controlled Trial with Parallel Design; RCTC: Randomized Controlled Trial with Cross-over Design; KT: Kinesio Taping; PT: Placebo Taping; NT: No Tape; PNF: Proprioceptive Neuromuscular Facilitation Stretching; SS: Static Stretching; RF: Rectus Femoris; VL: Vastus Lateralis; VM: Vastus Medialis; VMO: Vastus Medialis Oblique; HS: Hamstring; BF: Biceps Femoris; ST: Semitendinosus; SM: Semimembranosus; GN: Gastrocnemius

**Table S2** Characteristics of the included studies in population without disabilities

| **Author, (year)** | **Study design (washout period)** | **Intervention (N)** | **Age [year(SD)]** | **Gender (M, F)** | **Brief details of intervention** | **Outcome measures included** |
| --- | --- | --- | --- | --- | --- | --- |
| Baştürk, 2015^16^ | RCTC (Not indicated) | KT (18) NT (18) | 22.6(1.64) | KT (18, 0) NT (18, 0) | KT: Facilitation, 1 strips (Qs), 10-15% tension, Y-shaped NT: No tape | Vertical jump height |
| Cheung, 2016^17^ | RCTC (7 days) | KT (30) PT (30) | 17(2.6) | 12, 18 | KT: Facilitation, 2 strips (Qs, GN), 75% tension, I-shaped (Qs), Y-shaped (GN) PT: 2 strips (Qs, GN), no tension, I-shaped (Qs), Y-shaped (GN) | Counter-movement jump height |
| Dos Santos Glória, 2017^18^ | RCTP | KT (12) PT (12) | KT 17(0.9) PT 16.5(1.6) | 24, 0 | KT: Facilitation, 1 strip (RF), 15-20% tension, Y-shaped  PT: 1 strip (RF), no tension, Y-shaped | Isometric knee extension peak torque Distance in a single-leg hop |
| Fernandes de Jesus, 2016^19^ | RCTP | KT (20, 40 lower limbs) PT (18, 36 lower limbs) NT (19, 38 lower limbs) | KT 23.1(0.9) PT 24.6(2.6) NT 24.8(4.6) | 30, 30 | KT: Facilitation, 1 strip (RF), no tension, Y-shaped  PT: Transverse, 2 strips, no tension, I-shaped  NT: No tape | Isometric quadriceps strength Distance in a single-leg hop |

Continued

**Table S2** Continued

| **Author, (year)** | **Study design (washout period)** | **Intervention (N)** | **Age [year(SD)]** | **Gender (M, F)** | **Brief details of intervention** | **Outcome measures included** |
| --- | --- | --- | --- | --- | --- | --- |
| de Jesus, 2017^20^ | RCTP | KT 100 (26) KT 75 (26) KT 50 (20) KT 0 (20) NT (20) | KT 100 30.3(2.6) KT 75 29.5(1.4) KT 50 28.7(2.1) KT 0 29.2(1.8) NT 28.3(3.1) | KT 100 (13, 13) KT 75 (13, 13) KT 50 (8, 12) KT 0 (8, 12) NT (10, 10) | KT 100: Facilitation, 1 strip (RF), 100% tension, Y-shaped  KT 75: Facilitation, 1 strip (RF), 75% tension, Y-shaped  KT 50: Facilitation, 1 strip (RF), 50% tension, Y-shaped  KT 0: Facilitation, 1 strip (RF), no tension, Y-shaped  NT: No tape | Isometric quadriceps strength Distance in a single-leg hop |
| Fu, 2008^53^ | RCTC (7 days) | KT (14) NT (14) | 19.7(1.0) | 7, 7 | KT: Facilitation, 1 strip (RF), 120% length, Y-shaped NT: No tape | Isokinetic concentric knee extension peak torque (60°/s)  Isokinetic eccentric knee extension peak torque (60°/s) |
| Guedes, 2016^21^ | RCTP | KT (11) PT (10) | KT 20.1(1.9) PT 20.2(2.3) | 21, 0 | KT: Facilitation, 1 strip (Qs), 40% tension, I-shaped PT: 1 strip (Qs), no tension, I-shaped | Isokinetic concentric knee extension peak torque (60°/s) |
| Lee, 2017^22^ | RCTC (7 days) | KT (18) PT (18) NT (18) | 24.44(3.05) | 7, 11 | KT: Facilitation (RF), Inhibition (patella tendon), 4 strips (RF, patella tendon, superior & inferior meniscus), tension not indicated (RF & meniscus), 120% length (patella tendon), Y-shaped (RF & patella tendon), I-shaped (meniscus) PT: 3M tape, same taping method as KT NT: No tape | Isometric quadriceps strength |

Continued

**Table S2** Continued

| **Author, (year)** | **Study design (washout period)** | **Intervention (N)** | **Age [year(SD)]** | **Gender (M, F)** | **Brief details of intervention** | **Outcome measures included** |
| --- | --- | --- | --- | --- | --- | --- |
| Lins, 2013^54^ | RCTP | KT (20) PT (20) NT (20) | 23.3(2.5) | 0, 60 | KT: Facilitation, 3 strips (RF, VL, VM), 50% tension, I-shaped  PT: Non-elastic tape, 3 strips (RF, VL, VM), I-shaped  NT: No tape | Isokinetic concentric knee extension peak torque (60°/s)  Isokinetic eccentric knee extension peak torque (60°/s) Distance in a single-leg hop |
| Lins, 2016^23^ | RCTP | KT (12) PT (12) NT (12) | KT 23.3(3.1) PT 22.3(3.8) NT 21.4(3.6) | 0, 36 | KT: Facilitation, 3 strips (RF, VL, VM), 50% tension, I-shaped  PT: 3 strips (RF, VL, VM), no tension, I-shaped  NT: No tape | Isokinetic concentric knee extension peak torque (60°/s) Distance in a single-leg hop |
| Magalhães, 2016a^24^ | RCTC (2 days) | FKT (20) IKT (20) NT (20) | 24.7(4.4) | 20, 0 | FKT: Facilitation, 2 strips (QN), 30% tension, Y-shaped IKT: Inhibition, 2 strips (QN), 30% tension, Y-shaped NT: No tape | Isometric ankle plantar-flexion peak torque |
| Magalhães, 2016b^25^ | RCTP | KT (11) PT (10) | KT 20.91(2.23) PT 21.80(2.22) | 21, 0 | KT: Facilitation, 1 strip (RF), 40% tension, I-shaped PT: 1 strip (RF), no tension, I-shaped | Distance in a single-leg hop Single leg vertical jump height |
| Mostaghim, 2016^26^ | RCTC (7 days) | KT (44) NT (44) | 18 to 29 | 23, 21 | KT: Facilitation, 1 strip (Qs), 15-25% tension, Y-shaped NT: No tape | Isometric knee extension peak torque Sargent vertical jump height |

Continued

**Table S2** Continued

| **Author, (year)** | **Study design (washout period)** | **Intervention (N)** | **Age [year(SD)]** | **Gender (M, F)** | **Brief details of intervention** | **Outcome measures included** |
| --- | --- | --- | --- | --- | --- | --- |
| Nunes, 2013^55^ | RCTC (2 days) | KT (20) PT (20) | 22.3(3.3) | 11, 9 | KT: Facilitation, 1 strip (GN), 50% tension, Y-shaped PT: Non-elastic adhesive tape, same taping method as KT | Counter-movement jump height |
| Poon, 2015^27^ | RCTC (7 days) | KT (30) PT (30) NT (30) | 21.8(3.05) | 12, 18 | KT: Facilitation, 2 strips (RF, VM), 35% tension, Y-shaped PT: 2 strips (RF, VM), no tension, Y-shaped NT: No tape | Isokinetic concentric knee flexion peak torque (60°/s) |
| Serra, 2015^28^ | RCTP | KT (16) PT (18) | 23(1) | 20, 14 | KT: Facilitation, 1 strip (Qs), tension not indicated, V-shaped PT: 3M Micropore, same taping method as KT | Isometric knee extension peak torque |
| Vercelli, 2012^56^ | RCTC (7 days) | FKT (34) IKT (34) PT (34) | 23(5) | 17, 19 | FKT: Facilitation, 1 strip (Qs), 25-50% tension, Y-shaped IKT: Inhibition, 1 strip (Qs), 15-25% tension, Y-shaped PT: Transverse, 1 strip, no tension, I-shaped | Isokinetic concentric knee flexion peak torque (60°/s) |
| Vithoulk, 2010^2^ | RCTC (3 days) | KT (20) PT (20) NT (20) | 27(3.77) | 0, 20 | KT: Facilitation, 3 strips (RF, VL, VM), tension not indicated, Y-shaped (RF), I-shaped (VL, VM) PT: Transverse, 2 strips, I-shaped NT: No tape | Isokinetic concentric knee extension peak torque (60°/s)  Isokinetic eccentric knee extension peak torque (60°/s) |
| Wilson, 2016^29^ | RCTP | KT (8) PT (9) | KT 22.00(1.58) PT 24.63(5.85) | 9, 8 | KT: Facilitation, 1 strip (GN), 50% tension, Y-shaped PT: 1 strip (GN), no tension, Y-shaped | Distance in a single-leg hop |
| Wong, 2012^57^ | RCTC (7 days) | KT (30) NT (30) | 28.4(4.7) | 14, 16 | KT: Facilitation, 1 strip (VM), 75% tension, Y-shaped NT: No tape | Isokinetic concentric knee extension peak torque (60°/s) |

Continued

**Table S2** Continued

| **Author, (year)** | **Study design (washout period)** | **Intervention (N)** | **Age [year(SD)]** | **Gender (M, F)** | **Brief details of intervention** | **Outcome measures included** |
| --- | --- | --- | --- | --- | --- | --- |
| Yeung, 2016^4^ | RCTC (Not indicated) | FKT (28) IKT (28) PT (28) | 20 to 24 | 13, 15 | FKT: Facilitation, 1 strip (RF), 50% tension, Y-shaped IKT: Inhibition, 1 strip (RF), 15% tension, Y-shaped PT: Hypafix tape, same taping method as FKT | Isokinetic concentric knee extension peak torque (60°/s) |

RCTP: Randomized Controlled Trial with Parallel Design; RCTC: Randomized Controlled Trial with Cross-over Design; KT: Kinesio Taping; FKT: Facilitatory Kinesio Taping Application; IKT: Inhibitory Kinesio Taping Application; PT: Placebo Taping; NT: No Tape; Qs: Quadriceps Femoris; RF: Rectus Femoris; VL: Vastus Lateralis; VM: Vastus Medialis; GN: Gastrocnemius

**Table S3** Characteristics of the included studies in population with chronic musculoskeletal diseases

| **Author, Year (Condition)** | **Study design** | **Intervention (N)** | **Age [year(SD)]** | **Gender (M, F)** | **Brief details of intervention** | **Outcome measures included** |
| --- | --- | --- | --- | --- | --- | --- |
| Anandkumar, 2014^30^ (Knee osteoarthritis) | RCTP | KT (20) PT (20) | KT 55.7(5.8) PT 55.9(5.0) | KT (9, 11) PT (8, 12) | KT: Facilitation, 3 strips (RF, VL, VM), 50-75% tension, I-shaped PT: 3 strips (RF, VL, VM), no tension, I-shaped | Isokinetic concentric knee extension peak torque (90°/s)  Isokinetic eccentric knee extension peak torque (90°/s) |
| Aydoğdu, 2017^31^ (Knee osteoarthritis) | RCTP | KT + CT (28) CT (26) | KT + CT 52.53(9.68) CT 51.19(8.94) | 8, 46 | KT + CT : Facilitation, 2 strips (RF, HS), 50-70% tension, Y-shaped + Conventional treatment CT: Conventional treatment | Isometric quadriceps strength |
| Aytar, 2011^58^ (Patellofemoral pain syndrome) | RCTP | KT (12) PT (10) | KT 22.41(1.62) PT 26.20(3.52) | 0, 22 | KT: Facilitation, 2 strips (Qs), 10-15% tension, Y-shaped; 2 strips (Patella), 50-75% tension, I-shaped PT: Sticking plaster, same taping method as KT | Isokinetic concentric knee extension peak torque (60°/s) |
| Dhanakotti, 2015^32^ (Knee osteoarthritis) | RCTP | KT + CT (15) CT (15) | KT + CT 51.73 (5.10) CT 51.26(4.86) | KT + CT (4, 11) CT (3, 12) | KT + CT: Facilitation, 2 strip (RF, VM), 40% tension, Y-shaped + Conventional treatment CT: Conventional treatment | Isometric quadriceps strength |
| Kaya Mutlu, 2017^33^ (Knee osteoarthritis) | RCTP | KT (20) PT (19) | KT 54.25(6.01) PT 57.10(6.26) | KT (4, 16) PT (2, 17) | KT: Facilitation, 2 strips (Qs, HS), 25% tension (Qs), HS tension not indicated, Y-shaped PT: Transverse, 4 strips (Qs, HS), I-shaped | Isometric knee extension peak torque |

Continued

**Table S3** Continued

| **Author, Year (Condition)** | **Study design** | **Intervention (N)** | **Age [year(SD)]** | **Gender (M, F)** | **Brief details of intervention** | **Outcome measures included** |
| --- | --- | --- | --- | --- | --- | --- |
| Kim, 2017^34^ (Knee pathology history) | RCTP | KT (8) PT (8) | KT 25(5.0) PT 23(3.5) | KT (7, 1) PT (2, 6) | KT: Facilitation, 1 strip (RF), 25% tension, Y-shaped PT: Non-elastic tape, 1 strip (RF), Y-shaped | Isometric knee extension peak torque |

RCTP: Randomized Controlled Trial with Parallel Design; KT: Kinesio Taping; PT: Placebo Taping; CT: Conventional Treatment; Qs: Quadriceps Femoris; RF: Rectus Femoris; VL: Vastus Lateralis; VM: Vastus Medialis; HS: Hamstring

**Table S4** Characteristics of the included studies in population under post-operative orthopaedic conditions

| **Author, Year (Condition)** | **Study design** | **Intervention (N)** | **Age [year(SD)]** | **Gender (M, F)** | **Brief details of intervention** | **Outcome measures included** |
| --- | --- | --- | --- | --- | --- | --- |
| Balki, 2016^35^ (4 days post-ACLR) | RCTP | KT + CT (15) PT + CT (15) | KT + CT 28.60(4.50) PT + CT 27.66(7.45) | KT + CT (15, 0) PT + CT (15, 0) | KT + CT: Facilitation, 2 strips (RF, HS), 25-30% tension (RF), 40-50% (HS), Y-shaped; Lymphatic correction, 2 strips (Lymph node), 15% tension, fan-shaped + Rehabilitation program PT + CT: Transverse, 2 strips, no tension, I-shaped + Rehabilitation program | Isometric quadriceps strength |
| Balki, 2017^36^ (4 days post-ACLR) | RCTP | KT + CT (13) PT + CT (13) | KT + CT 27.7(4.1) PT + CT 27.1(7.5) | KT + CT (13, 0) PT + CT (13, 0) | KT + CT: Facilitation, 2 strips (RF, HS), 30-45% tension, Y-shaped; Lymphatic correction, 2 strips (Lymph node), 15% tension, fan-shaped + Rehabilitation program PT + CT: Transverse, 2 strips, no tension + Rehabilitation program | Isometric hip flexor strength |
| Harput, 2016^37^ (6 months post-ACLR with Tampa Kinesiophobia) | RCTC (7 days) | KT (30) NT (30) | 25.1(7.8) | Not indicated | KT: Facilitation, 2 strips (RF, patella tendon), no tension (RF), 100% tension (patella tendon), Y-shaped  NT: No tape | Isokinetic concentric knee extension peak torque (60°/s) Distance in a single-leg hop |
| Oliveira, 2016^38^ (12 to 17 weeks post-ACLR) | RCTP | KT (15) PT (15) NT (15) | 28.6(3.8) | KT (15, 0) PT (15, 0) NT (15, 0) | KT: Facilitation, 3 strips (RF, VL, VM), 50% tension, I-shaped PT: 3 strips (RF, VL, VM), no tension, I-shaped NT: No tape | Isokinetic concentric knee extension peak torque (60°/s)  Isokinetic eccentric knee extension peak torque (60°/s) |

ACLR: Anterior Cruciate Ligament Reconstruction; RCTP: Randomized Controlled Trial with Parallel Design; RCTC: Randomized Controlled Trial with Cross-over Design; KT: Kinesio Taping; PT: Placebo Taping; NT: No Tape; CT: Conventional Treatment; RF: Rectus Femoris; VL: Vastus Lateralis; VM: Vastus Medialis; HS: Hamstring

**Table S5** PEDro scale scoring

| **Studies** | **Q1** | **Q2** | **Q3** | **Q4** | **Q5** | **Q6** | **Q7** | **Q8** | **Q9** | **Q10** | **Q11** | **Total** |
| --- | --- | --- | --- | --- | --- | --- | --- | --- | --- | --- | --- | --- |
| Ahn 2015 | 0 | 1 | 0 | 1 | 1 | 0 | 0 | 1 | 0 | 1 | 1 | 6 |
| Anandkumar 2014 | 1 | 1 | 1 | 1 | 0 | 0 | 1 | 1 | 1 | 1 | 1 | 8 |
| Aydoğdu 2017 | 1 | 1 | 1 | 1 | 0 | 0 | 0 | 1 | 0 | 1 | 1 | 6 |
| Aytar 2011 | 1 | 1 | 0 | 1 | 1 | 0 | 1 | 1 | 0 | 1 | 1 | 7 |
| Balki 2016 | 0 | 1 | 0 | 1 | 0 | 0 | 0 | 1 | 0 | 1 | 1 | 5 |
| Balki 2017 | 0 | 1 | 0 | 1 | 1 | 0 | 1 | 1 | 0 | 1 | 1 | 7 |
| Baştürk 2015 | 0 | 1 | 0 | 0 | 0 | 0 | 0 | 0 | 0 | 1 | 1 | 3 |
| Boobphachart 2017 | 0 | 1 | 0 | 1 | 0 | 0 | 0 | 0 | 0 | 1 | 1 | 4 |
| Boozari 2018 | 0 | 1 | 0 | 0 | 0 | 0 | 0 | 1 | 0 | 1 | 1 | 4 |
| Chen 2013 | 1 | 1 | 0 | 1 | 0 | 0 | 0 | 0 | 0 | 1 | 1 | 4 |
| Cheung 2016 | 1 | 1 | 0 | 0 | 1 | 0 | 0 | 0 | 0 | 1 | 1 | 4 |
| Dhanakotti 2015 | 1 | 1 | 0 | 1 | 0 | 0 | 1 | 1 | 0 | 1 | 1 | 6 |
| Dos Santos Glória 2017 | 0 | 1 | 1 | 1 | 1 | 0 | 0 | 0 | 0 | 1 | 1 | 6 |
| Fernandes de Jesus 2016 | 1 | 1 | 1 | 1 | 0 | 0 | 1 | 1 | 0 | 1 | 1 | 7 |
| de Jesus 2017 | 1 | 1 | 0 | 1 | 0 | 0 | 1 | 1 | 0 | 1 | 1 | 6 |
| Fu 2008 | 1 | 1 | 0 | 0 | 0 | 0 | 0 | 1 | 0 | 1 | 0 | 3 |
| Guedes 2016 | 1 | 1 | 1 | 1 | 1 | 0 | 0 | 0 | 0 | 1 | 1 | 6 |
| Haksever 2016 | 0 | 1 | 0 | 1 | 0 | 0 | 0 | 0 | 0 | 1 | 1 | 4 |
| Harput 2016 | 0 | 1 | 0 | 0 | 0 | 0 | 0 | 0 | 0 | 1 | 1 | 3 |
| Continued | | | | | | | | | | | | |

**Table S5** Continued

| **Studies** | **Q1** | **Q2** | **Q3** | **Q4** | **Q5** | **Q6** | **Q7** | **Q8** | **Q9** | **Q10** | **Q11** | **Total** |
| --- | --- | --- | --- | --- | --- | --- | --- | --- | --- | --- | --- | --- |
| Kaya Mutlu 2017 | 1 | 1 | 1 | 1 | 0 | 0 | 1 | 1 | 1 | 1 | 1 | 8 |
| Kim 2017 | 1 | 1 | 0 | 1 | 0 | 0 | 1 | 1 | 0 | 1 | 1 | 6 |
| Lee 2017 | 1 | 1 | 1 | 0 | 1 | 0 | 1 | 1 | 1 | 1 | 1 | 8 |
| Lins 2013 | 1 | 1 | 0 | 0 | 0 | 0 | 0 | 0 | 0 | 1 | 1 | 3 |
| Lins 2016 | 0 | 1 | 0 | 1 | 0 | 0 | 0 | 1 | 0 | 1 | 1 | 5 |
| Magalhães 2016a | 0 | 1 | 0 | 0 | 1 | 0 | 0 | 0 | 0 | 1 | 1 | 4 |
| Magalhães 2016b | 1 | 1 | 1 | 1 | 1 | 0 | 0 | 0 | 0 | 1 | 1 | 6 |
| Mostaghim 2016 | 1 | 1 | 0 | 0 | 0 | 0 | 0 | 0 | 0 | 1 | 0 | 2 |
| Nunes 2013 | 1 | 1 | 1 | 0 | 1 | 0 | 1 | 1 | 1 | 1 | 1 | 8 |
| Oliveira 2016 | 0 | 1 | 0 | 0 | 0 | 0 | 0 | 1 | 0 | 1 | 1 | 4 |
| Poon 2015 | 0 | 1 | 0 | 0 | 1 | 0 | 0 | 0 | 0 | 1 | 1 | 4 |
| Serra 2015 | 1 | 1 | 0 | 1 | 0 | 0 | 0 | 1 | 0 | 1 | 1 | 5 |
| Vercelli 2012 | 0 | 1 | 0 | 1 | 0 | 0 | 1 | 0 | 0 | 1 | 1 | 5 |
| Vithoulk 2010 | 0 | 1 | 0 | 0 | 0 | 0 | 0 | 0 | 0 | 1 | 1 | 3 |
| Wilson 2016 | 0 | 1 | 0 | 1 | 1 | 0 | 1 | 0 | 0 | 1 | 1 | 6 |
| Wong 2012 | 1 | 1 | 0 | 0 | 0 | 0 | 0 | 0 | 0 | 1 | 1 | 3 |
| Yeung 2015 | 0 | 1 | 1 | 1 | 0 | 0 | 1 | 1 | 0 | 1 | 1 | 7 |
| Yeung 2016 | 0 | 1 | 1 | 0 | 1 | 0 | 0 | 0 | 0 | 1 | 1 | 5 |

Q1: Eligibility criteria; Q2: Random allocation; Q3: Concealed allocation; Q4: Baseline comparability; Q5: Blind subjects; Q6: Blind therapists; Q7: Blind assessors; Q8: Adequate follow-up; Q9: Intention-to-treat analysis; Q10: Between-group comparisons; Q11: Point estimates and variability

**Table S6** Search strategy for EMBASE

| **#** | **Searches** |
| --- | --- |
| 1 | (athletic tap* or (kinesio* adj3 tap*) or kinesiotap* or (elastic adj2 tap*)).af. |
| 2 | (muscle or strength or peak torque or function or performance or hop or jump).af. |
| 3 | crossover-procedure/ or double-blind procedure/ or randomized controlled trial/ or single-blind procedure/ or (random* or factorial* or crossover* or cross over* or placebo* or (doubl* adj blind*) or (singl* adj blind*) or assign* or allocat* or volunteer*).tw. |
| 4 | 1 and 2 and 3 |
| 5 | limit 4 to (human and english language and yr="2007 - 2018") |

**Table S7** Search strategy for MEDLINE

| **#** | **Searches** |
| --- | --- |
| 1 | (athletic tap* or (kinesio* adj3 tap*) or kinesiotap* or (elastic adj2 tap*)).af. |
| 2 | (muscle or strength or peak torque or function or performance or hop or jump).af. |
| 3 | ((randomized controlled trial or controlled clinical trial).pt. or randomized.ab. or randomised.ab. or placebo.ab. or drug therapy.fs. or randomly.ab. or trial.ab. or groups.ab.) not (exp animals/ not humans.sh.) |
| 4 | 1 and 2 and 3 |
| 5 | limit 4 to (english language and humans and yr="2007 - 2018") |

**Table S8** Search strategy for CINAHL

| **#** | **Searches** |
| --- | --- |
| S1 | TX athletic tap* OR TX kinesio* n3 tap* OR TX kinesiotap* OR TX elastic n2 tap* |
| S2 | TX muscle OR TX strength OR TX peak torque OR TX function OR TX performance OR TX hop OR TX jump |
| S3 | S1 AND S2   - Limiters - Published Date: 20070101-20181231; English Language; Human; Randomized Controlled Trials |

**Table S9** Search strategy for CENTRAL

| **#** | **Searches** |
| --- | --- |
| 1 | (athletic tap* or (kinesio* adj3 tap*) or kinesiotap* or (elastic adj2 tap*)).af. |
| 2 | (muscle or strength or peak torque or function or performance or hop or jump).af. |
| 3 | 1 and 2 |
| 4 | limit 3 to (yr="2007 - 2018" and english language) |
